# Radiocarbon dating of wooden beams at Herodium palace-fortress: Modeling tree-felling events and their implications on site chronology

**DOI:** 10.1371/journal.pone.0352580

**Published:** 2026-10-15

**Authors:** Lior Regev, Roi Porat, Avner Botosh, Yakov Kalman, Johanna Regev, Eugenia Mintz, Uzi Leibner, Elisabetta Boaretto

**Affiliations:** 1 D-REAMS Radiocarbon Laboratory, Scientific Archaeology Unit, Weizmann Institute of Science, Rehovot, Israel; 2 Institute of Archaeology, The Hebrew University of Jerusalem, Jerusalem, Israel; University of South Florida, ITALY

## Abstract

The site of Herodium is best known for its impressive, well-preserved palace fortress, built by Herod the Great, towards the end of the 1^st^ century BC. The site's conical shape, artificially created to transform it into Herod’s monumental burial complex, is clearly seen from afar. The impressive preservation of wooden beams and posts at the site has allowed for a rigorous radiocarbon study of the beams’ felling dates and their potential implications for the timing of construction activities at the site. A total of 24 ^14^C measurements were performed on specific growth rings from 11 beams and posts, associated with six (sub) phases at the site. This level of detail, combined with Bayesian modeling and sensitivity testing of potential seasonal offsets, has revealed that the wood used at the site originates from four main tree-felling events. Apparently, the first event might have been as early as Alexander Jannaeus's time, in the first quarter of the 1^st^ century BC – a period not currently identified in the archaeological record at the site. The second reflects an extensive supply of wood, felled during the period corresponding to the beginning of the main phase of Herod’s massive construction activity (40–30 BC, approximately), followed by a possible third felling event towards the end of Herod’s life or shortly after. The fourth felling event was identified in beams used during the Bar-Kokhba revolt in 132–136 AD. However, the beams were felled several decades earlier, around 100 AD, between the Great Revolt (66–71 AD) and the Bar-Kokhba one, in a period characterized by limited documented occupation, indicating that the wood was used several decades after the trees were felled. The origin of some of those beams is probably from the extensive gardens Herod planted at the site.

## Introduction

The Palace of Herodium was constructed in several stages by King Herod the Great during his reign (37−4 BC), approximately 10 km south of Jerusalem, on a natural hill that had previously served as a stronghold in the Hellenistic period [1]. The main features of the Herod construction enterprise include a palace, a fortress, an administrative center, and a royal burial complex [2,3]. A fortified, circular palace was built at the hilltop, surrounded by a double wall with four towers (Fig 1). A royal theater, mausoleum, and storage rooms were built on the northern slope. Another building complex was excavated at the foot of the hill to the north, dubbed as “Lower Herodium,” where additional palace structures, an administrative center, and a central garden – the “pool garden” watered through an aqueduct from the west, were discovered. Toward the end of Herod's reign, the mountain and the royal buildings on it were converted into a burial mound. The entrance was blocked, and the natural mountain slopes were altered by quarrying and filling, forming a steep, conical, volcano-like shape [4].

**Fig 1:**
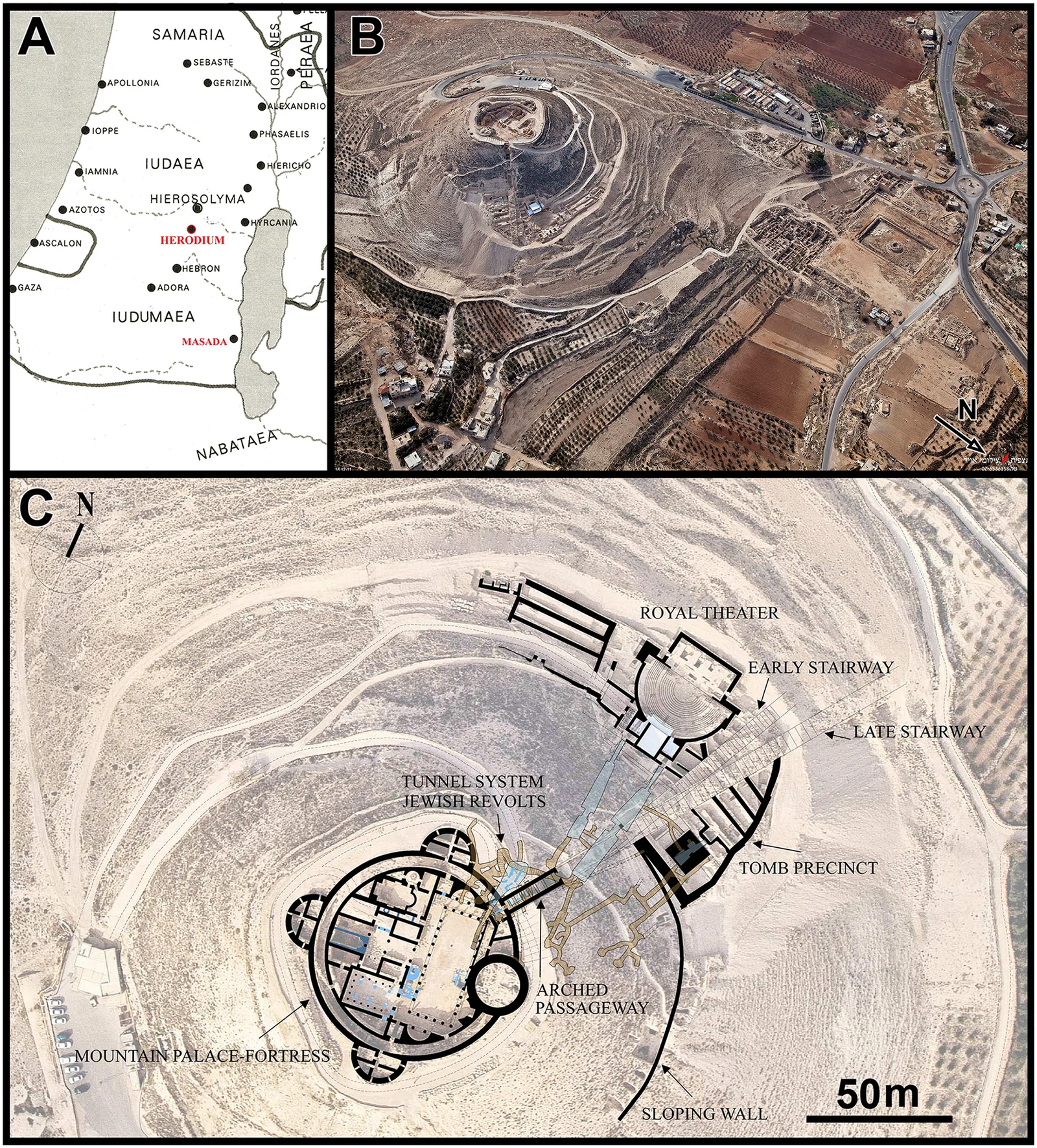
A. Location map of Herodium among the region's main settlements of the Herodian period. B and C. An image and a general plan of the site, respectively.

Following Herod’s death in 4 BC, Herodium kept its central administrative role in the region. Still, the activity at the site was primarily concentrated in the lower part, while the circular palace complex was largely neglected [5,6]. Decades later, during the Great Revolt, the Jewish rebels held the site until the Romans’ takeover in 71 AD. Understandably, the rebels chose to entrench themselves mainly within the Mountain Palace-Fortress, as evident from the remains of this period within the building, which included residences, a synagogue, and water facilities, using the Herodian building materials in secondary uses [7,8].

During the period between the Great Revolt and the Bar-Kokhba Revolt (132–136 AD), there are sparse remains within the fortified palace, possibly originating from a temporary Roman garrison or the re-established administrative function of the site, as Herodium was part of the capital of the Roman toparchy [6,9,10: Mur. 115]. During the Bar-Kokhba revolt, the fortress housed a headquarters of the rebels and their administrative center. The rebels built a vast assault and escape tunneling system used in their battle against the Romans until their defeat by the latter, who burnt the site, causing significant collapses [11–14]. The site remained unoccupied until a small monastery was built during the Byzantine period, and was finally deserted during the 9^th^ century AD [15].

Six major construction phases have been identified so far at the site based on stratigraphy, some are divided into sub-phases (Table 1).

**Table 1:** Construction phases (or sub-phases) at Herodium.

Phase No.	Historical period	Years
1a	Hellenistic period —first stronghold atop the hill	3^rd^–mid-2^nd^ century BC
1b*	Prior to Herod's main construction activities	40−25 BC
2a*	Early Herod's 1^st^ major construction activities	30−15 BC
2b*	Late Herod's architectural alterations	15−4 BC
3a,b*	Late Herod's artificial mount construction	15−4 BC
3c*	Late Herodian, post-artificial mount construction	10BC-66 AD
4	The Great Revolt	66-71 AD
5*	Bar-Kokhba Revolt	132-136 AD
6	The Byzantine Period	5^th^-9^th^ centuries AD

The phases are based on archaeological stratigraphy and historical evidence. Beams from the phases marked by an asterisk were sampled in the current study.

Compared to most hill country sites in Israel, the state of preservation of the timbers at the various structures of Herodium is outstanding. This is likely to be attributed to several proxies, most prominently the rapid intentional coverage of the structure, as well as the relatively drier climate at the site. Wood was used in many locations in the buildings, such as on floors, walls, roofs, door posts, and installations [16]. While the main building material was stone and the bonding agent, wood was incorporated to enhance constructional strength, stability, and aesthetic beauty.

The abundance of wood created a unique opportunity to use radiocarbon dating to assess how many tree-felling events are represented in the wooden elements associated with the site’s construction history, mainly during the Herodian period through the Bar-Kokhba revolt in the second century AD. Were the trees felled in a separate event for each new building episode, or do they originate from one or a few felling events, in which case they were stored and used for construction from storage?

We assume that most of the trees needed for the main construction projects were felled in close time proximity to their shipment and primary use at the site, as seen in other studies [17,18]. Secondary use of wooden beams is possible, of course. However, due to the abundance of wood at the site and the large building scale, we believe that ordered shipments were needed.

Although hundreds of wood remains were found at the site, only a handful are logs with more than c. 30 growth rings. Therefore, dendrochronological dating methods were not applicable, and radiocarbon dating was applied.

The last 14,000 years or so of the radiocarbon calibration curve are based on tree rings, which have been independently dated by dendrochronology [19]. In recent years, there has been a growing effort to improve the calibration resolution by dating single rings, as opposed to chunks of 10 and 20 rings that were dated together until a decade or two ago [19–27]. Since the curve resolution during the relevant period (1^st^ century BC – 2^nd^ century AD) was mainly built using measurements of sections of 10 rings (63 sections of 10 rings, 16 of 20 rings, and 3 of 29–31 rings, www.intcal.org), we rounded all the calibrated results by 10 years (see Materials and Methods).

IntCal20, the calibration curve for the Northern Hemisphere, which is used in this study, is constructed primarily from European and American tree-ring measurements [19]. However, high-precision studies reveal that the curve does not always accurately capture local conditions. Small but sometimes significant offsets have been documented in the Mediterranean, where they can translate into discrepancies of several decades [28–30]. These offsets likely reflect differences in growing seasons — with some Mediterranean plants recording atmospheric ^14^C in winter and spring rather than the summer-dominated growth of European oaks and American pines used for IntCal20. That is the case for samples at the Eastern Mediterranean region of the Southern Levant and Egypt, where offsets from the standard calibration curve were found [25,29–32] in the recently updated magnitude of 11.3 ± 5.2 BP [33]. However, some of the above studies further found that the offset magnitude is not constant and varies in time [28,33].

As most of the wood in Herodium is cypress wood [34,35], a growing-season radiocarbon offset should be considered. Cypress is a natural component of the Mediterranean maquis forest. Its archaeological abundance had increased significantly in the Early Roman period [34,36], and it is believed to grow in the Judean region of Jerusalem based on historical sources and high pollen concentrations extracted from plaster from a Herodian structure in Jerusalem [36].

In the southern Levant, cypress grows in the rainy season, e.g., during the autumn, winter, and spring [37,38]. Hence, its radiocarbon content is expected to present a seasonal offset.

## Materials and methods

### Archaeological excavation

The Hebrew University expedition to Herodium has been conducting excavations at the site for approximately fifty years, under annual permits lawfully issued by the Israeli Staff Officer for Archaeology. The permit numbers relevant for this study are 04-01-2019, 01-01-2020, and 06-01-2022. All necessary permits were obtained for the described study, and the study complied with all relevant regulations.

### Wood sampling and preparation

Most wooden beams and poles were preserved enough to be cored. The coring was performed using Dry Wood borers (Rinntech, Germany), 8 mm inner diameter, powered by a Makita BHP458 electric drill. Cores were fastened to a wooden rack without glue (the depression of the rack can be widened and narrowed using screws). The cores were sanded to visualize the growth rings better using an orbit sander with sandpaper discs gradually increasing from 150 to 1000 grit size. Wherever coring was not possible, the outmost ring was sampled in the field. The number of rings was counted under a binocular (M80, Leica). Due to the low number of rings (30 or less), ring width measurements and cross-dating were not performed.

### Botanical identification

The botanical identification was made using a Nikon Eclipse LV150N microscope by comparing to the Kimmel Center for Archaeological Science reference collection and using wood anatomy atlases [39,40].

### α-cellulose extraction

20–40 mg of a single ring (unless stated otherwise) were placed in 16 x 125 mm borosilicate glass test tubes. The tops of the tubes were stuffed with meshed glass wool. The pretreatment starts with only the first two steps out of the standard Acid-base-acid (ABA) pretreatment, which is based on Southon and Magana [41]. The third acid step is the first step in the bleaching procedure that follows. In short, samples were treated with aliquots of 5 ml 1N HCl for 1 hour, washed with DDW, and then 5 ml of 0.1N NaOH were added for 1 hour, followed by washing with DDW. Finally, the samples were treated for 1 hour with 5 ml 1N HCl in a water bath at 70 °C to remove any carbon that may have adhered during the former alkaline treatment. Then, α-cellulose was extracted using a modification to a variation of the Jayme-Wise method suggested by Southon and Magana [41]: 2.5 ml of 1N HCl and 2.5 ml of 1M NaClO_2_ were added to the samples, which were then transferred to a water bath at 70 °C for 1 hour. If additional bleaching was needed, the chemicals were replaced by fresh ones for an additional 1 hour. After bleaching, the samples were washed with DDW. Next, the samples were treated with 6 ml of 5N NaOH for 1 hour, followed by washing with DDW and subsequently treated for 1 hour with 5 ml of 1N HCl in a water bath at 70 °C. The samples were then washed with DDW until reaching a neutral pH and dried in an oven at 100 °C.

### Radiocarbon dating and modeling

2–4 mg of α-cellulose were weighed into tin capsules (8X5 mm, ‘Elemental Microanalysis’) and burnt in an elemental analyzer. Graphitization and stable isotopes measurement was performed using an EA-IRMS-AGE3 system, composed of the elemental analyzer (EA, ‘vario ISOTOPE SELECT’ by Elementar), coupled to an isotope ratio mass spectrometer (IRMS, ‘isoprime precisION’ by Elementar) and a third generation of the automated graphitization equipment (AGE3, Ionplus) [42]. Carbon percentages were measured by the EA, and the δ^13^C values by the IRMS. The graphite was then pressed into an aluminum cathode, and the ^14^C content was measured at the DANGOOR Research Accelerator Mass Spectrometry Laboratory at the Weizmann Institute of Science [43]. The radiocarbon age was then modeled by an ‘outlier model’ [44] and calibrated in OxCal 4.4.2 or 4.4.4 software package [45] with IntCal20 calibration curve [19] to obtain the calendar date. Wherever several rings from the same tree were dated, ‘wiggle-matching’ was performed using the V_Sequence function to include an uncertainty in the gap between each two measurements [46]. Due to the resolution of the calibration curve in the relevant period for this study, we rounded the calibrated results by 10 years, using OxCal (Edit => Format => Round by 10). Whenever an offset was applied, the Delta_R function was used with 11 ± 5 for cypress and pine samples, and 0 ± 0 for cedar and fir.

Since our samples are highly controlled, the calibrated and modeled ranges reported in the text are the 68.3% probability, unless otherwise mentioned. Three main parameters affect sample accuracy and precision [47]. The first is the archaeological context, which is well-defined in this study. The authors did the field sampling in a collaboration between the wood and radiocarbon specialists and the site’s archaeologists. The second parameter is the suitable pretreatment protocol, and the third is the measurement itself. We have complete control over the process, as all samples were pretreated and measured in the D-REAMS lab. We follow parameters like the pretreatment efficiency, %C within the cleaned sample, and δ^13^C of the cellulose (Table 2) to verify the wood's pretreatment quality and preservation state. In the measurement, we routinely use 7 oxalic acid standards and four known age standards in each 40-sample wheel for improved assessment of the AMS operation [43]. We choose to use the 68.3% probabilities in our chronologies since we believe we have very good control over the complete field-to-date process, which minimizes the number of outlier dates (one outlier out of 24 dates in this study).

**Table 2:** Radiocarbon measurements and modeled calibrated ages. Samples are grouped according to the archaeological construction phases with which they are associated. The radiocarbon results of the outermost rings of each sample represent its felling time (or slightly earlier where outer rings are missing) and should be considered as terminus post quem for the associated construction activity.

Construction Phase	Sample No.	Sample Name and Description	Taxonomy	C %	Libby Age [unCalBP]	Modeled Calibrated (68.3%)	Modeled Calibrated (95.4%)	AMS δ^13^C [‰]	IRMS δ^13^C [‰]
1b: Prior to Herod's main construction activities	RTD 12280	HRD22−21 Pith	Cupressus sempervirens	44	2089 ± 15	130BC(68.3%)110BC	150BC(95.4%)100BC	−19.03	
	RTD 12281	HRD22−21 18^th^ from bark and 25^th^ rings from pith		44	2077 ± 15	110BC (68.3%) 90BC	120BC (95.4%) 80BC	−18.60	
	**RTD 12282**	**HRD22−21** **Rings 40 & 41 from pith; 3**^**rd**^ **and 4**^**th**^ **rings from bark**		**44**	**2038 ± 39**	**90BC (68.3%) 70BC**	**110BC (95.4%) 60BC**	**−20.96**	
	RTD 12039*	HRD22−21 2 outermost rings		43	1981 ± 24	Outlier. It was not part of the model.		−19.30	−19.63
	RTD 12283	HRD22−22 Innermost ring	Cupressus sempervirens	38	2129 ± 15	130BC(68.3%)100BC	140BC(95.4%)100BC	−21.12	
	**RTD 12040**	**HRD22−22** **Outermost 1**^**st**^ **to 4**^**th**^ **rings**		**38**	**2050 ± 23**	**100BC (64.2%) 70BC**	**110BC (95.4%) 70BC**	**−17.26**	**−19.89**
2a: Early Herod's 1^st^ major construction activities	**RTD 10489**	**HRD20-8B** **Outermost ring**	**Cupressus sempervirens**	**43**	**2005 ± 18**	**50BC (68.2%) 20BC**	**50BC (95.4%) 10BC**	**−18.10**	**−18.75**
	**RTD 10490.1**	**HRD20−9**	**Sample too small**	**43**	**1959 ± 29**	**R_Combine** **50BC (68.2%) 20BC**	**R_Combine** **50BC (95.4%) 10BC**	**−21.82**	**−21.65**
	**RTD 10490.2**				**2040 ± 38**			**−20.28**	**−21.25**
	**Combine**				**1989 ± 24**	**Χ** ^**2**^ **-Test: df = 1, T = 2.9 (5% is 3.8)**			
2b: Late Herod’s architectural alterations	RTD 12284	HRD20-10A Innermost ring	Cupressus sempervirens	44	2062 ± 21	80BC (68.3%) 60BC	90BC (95.4%) 50BC	−20.95	
	RTD 12285	HRD20-10A 10-14 rings from innermost one		43	2118 ± 16	70BC (68.3%) 50BC	80BC (95.4%) 40BC	−21.54	
	RTD 10643	**HRD20-10A Outermost**		**43**	**2047 ± 17**	**50BC (68.3%) 30BC**	**60BC (95.4%) 20BC**	**−20.77**	**−21.41**
3b: Late Herod’s artificial mount construction	RTD 10666	HRD20-13A Ring 2 from pith	Abies alba	43	2104 ± 17	70BC (68.3%) 50BC	80BC (95.4%) 50BC	−21.91	−21.84
	RTD 10667	HRD20-13A Ring 19 from pith		40	2061 ± 17	60BC (68.3%) 40BC	60BC (95.4%) 30BC	−21.12	−20.93
	**RTD 10491**	**HRD20-13A Outermost**		**43**	**2031 ± 19**	**50BC (68.3%) 30BC**	**50BC (95.4%) 20BC**	**−21.36**	**−21.90**
3c: Late Herodian, post artificial mount construction	**RTD 10492**	**HRD20-15a Outermost**	**Cupressus sempervirens**	**43**	**2020 ± 18**	**30BC (68.3%) 10AD**	**40BC (95.4%) 30AD**	**−23.14**	**−22.85**
5: Bar-Kokhba Revolt	**RTD 10304**	**HRD19-4B**	**Cupressus sempervirens**	**43**	**1939 ± 18**	**70AD (68.3%) 110AD**	**60AD (95.4%) 130AD**	**−20.66**	
	RTD 10335	HRD19-5A Ring 2 from pith	Pinus	37	1938 ± 28	60AD (68.3%) 90AD	50AD (95.4%) 100AD	−21.43	−21.40
	**RTD 10305.1**	**HRD19-5A** **Outermost rings**		**41**	**1951 ± 17**	**R_Combine** **90AD (68.3%) 120AD**	**R_Combine** **80AD (95.4%) 130AD**	**−20.15**	**−20.83**
	**RTD 10305**			**40**	**1912 ± 31**			**−25.13**	
	**Combine**				**1942 ± 15**	**Χ** ^**2**^ **-Test: df = 1, T = 1.2 (5% is 3.8)**			
	**RTD 10306**	**HRD19-6B** **Rings 13–14 from pith**	Cedrus libani	**42**	**1934 ± 28**	**80AD (68.3%) 120AD**	**70AD (95.4%) 130AD**	**−21.54**	**−21.25**
	RTD 10336	HRD19-6B Innermost ring		43	1905 ± 28	70AD (68.3%) 100AD	60AD (95.4%) 120AD	−21.99	−22.57
	RTD 11464	HRD20−18 Ring 3 from pith	Cupressus sempervirens	42	1911 ± 24	70AD (68.3%) 100AD	60AD (95.4%) 120AD	−21.83	−21.70
	**RTD 11465**	**HRD20−18** **Ring 15 from pith**		**42**	**1923 ± 24**	**80AD (68.3%) 120AD**	**70AD (95.4%) 130AD**	**−23.10**	**−22.58**

The results in the table are according to the most statistically probable model (Fig 2 and supporting information). Samples from the outermost ring(s) are in bold. Samples from the same log are shaded the same. Sample RTD12039 is marked with an asterisk. It was identified in all models as an outlier and therefore was not included in the models.

## Results

Twenty-four radiocarbon dates were performed on specific growth rings from eleven wooden beams/posts associated with phases: 1b, 2a, 2b, 3b, 3c, and 5 (Tables 1,2). In seven samples, 2–4 rings were dated to improve precision by “wiggle matching” (Fig 2).

**Fig 2:**
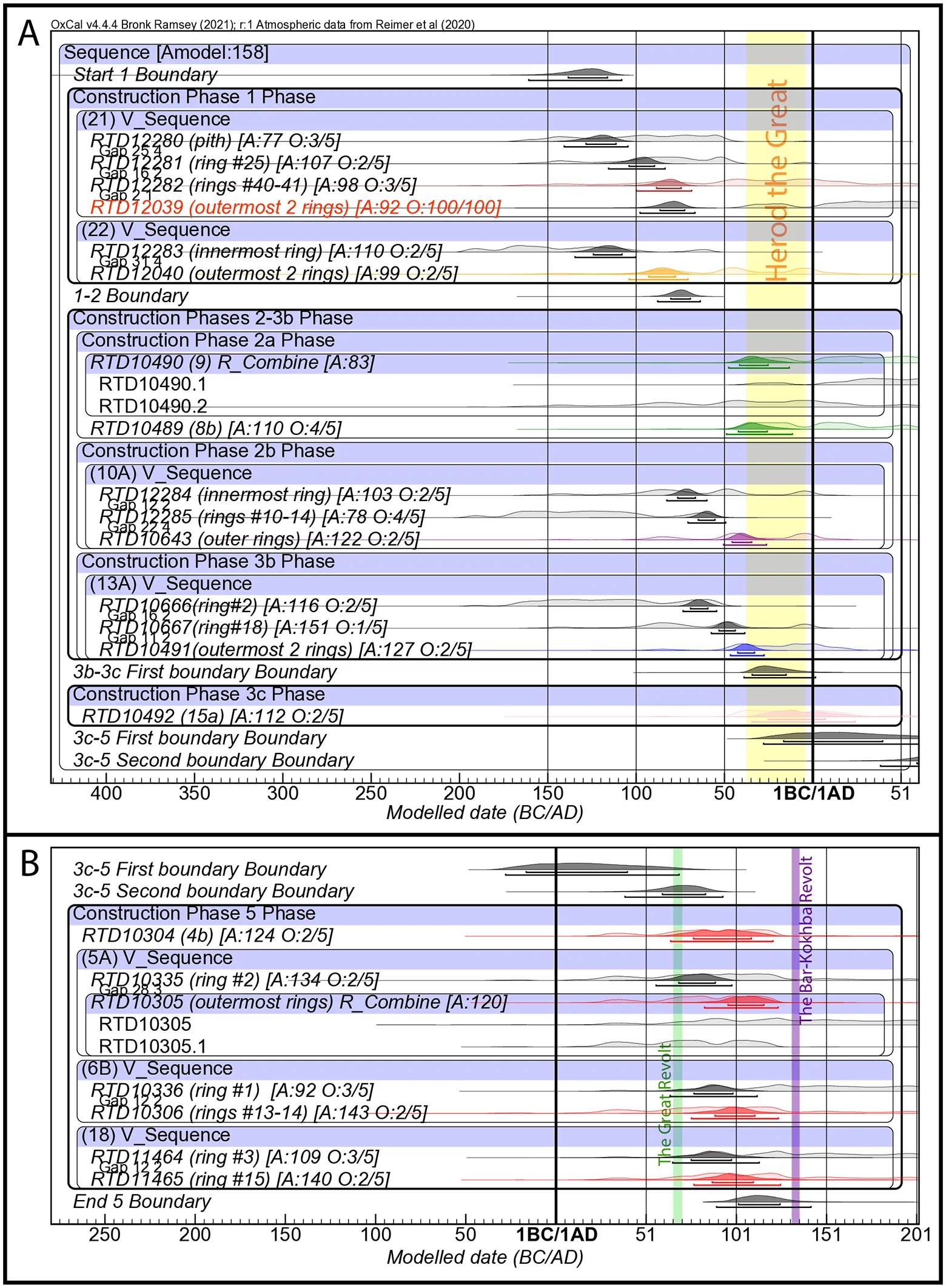
Bayesian modeling of the radiocarbon-dated samples. The numbers in parentheses refer to the sample number. For example, (9) is HRD20−9. The full results are in Table 2, and the model code is in S1 Text. Sample RTD 12039 (red font) was found to be an outlier and was set as such in the model (S2 Fig).

### The radiocarbon Bayesian model

Since all the samples are tree trunks or branches, the dates, representing the felling time, are all terminus post quem for the construction activity at which they were found. Accordingly, any interpretation linking the modeled felling events to specific construction activities relies on assumptions regarding the timing of wood use, including potential delays due to storage, transport, or reuse. However, a tree felled during the construction time of a certain phase could not have been used at an earlier phase.

To estimate the most probable number of tree-felling events, we tested four alternative chronological models. All models are based on 24 measurements from 11 beams, organized according to their stratigraphic associations as identified by the excavators, reflecting the site’s construction phases and modifications. The site has 10 archaeological (sub) phases that the excavators identified (Table 1). We sampled timbers associated with six of these separate phases.

Each model assesses a different number of felling events (2, 3, 4, or 6) and assesses their probability. The models were evaluated based on their overall agreement index (Amodel), the agreement values of individual samples (A), and the proportion of samples identified as potential outliers (O). The ‘preferred model’, comprising four felling events, was selected as it yields the highest overall agreement (Amodel:158, Fig 2) and does not require the exclusion of additional samples or the acceptance of elevated outlier probabilities, in contrast to the alternative models (Table 3; see S3–S5 Figs for the additional models). Hence, the results in this section are based on the preferred model, while the alternative models are discussed below.

**Table 3:** Statistical scores of models with and without an applied offset.

Model Name	Total Agreement (Amodel)	No. of Samples	
		A < 60	O > 5
2-Phase	118	0	10
**2-Phase Offsets**	**102**	**1**	**9**
3-Phase	116	1	9
3-Phase Offsets	100	1	11
**4-Phase**	**158**	**0**	**0**
4-Phase Offsets	80	3	1
6-Phase	12	3	4
6-Phase Offsets	13	4	4

‘Total agreement’ is the total statistical agreement score of the model. ‘A<60’ refers to the number of individual samples with an individual agreement below the 60 threshold. ‘O>5’ refers to the number of individual samples with an outlier probability above the 5% threshold. All models and their respective calibrated age plots are in the online supplementary information files.

In OxCal software, which utilizes Bayesian statistics to construct chronological models, the dates from each presumed felling event are aggregated in a Phase function, reflecting the assumption that they were felled around the same time. To distinguish the Phase function in the chronological models from the archaeological construction phase, the Phase function will be referred to as the ‘OxCal Phase’ throughout the paper.

In all models, when multiple rings were measured from a single log, they were placed in a V_Sequence with a gap based on the ring count. In cases where the ring count could not be established with absolute certainty or when the species dated was a cypress, known to sometimes produce false rings, we added a larger uncertainty to the gaps. Sample **RTD 12039** was identified as an analytical outlier. It appears in the models but is not considered in the analysis. See S2 Fig for details.

### Applying an offset

First, we tested the four models without applying an offset (Fig 2, and S3–S5 Figs). Then, to assess the sensitivity of the models to a potential seasonal radiocarbon offset, a ΔR value of 11 ± 5 BP [33] was applied to all cypress (Cupressus sempervirens) and pine (Pinus) samples (see S6–S9 Figs, and Text S1for all OxCal models). Interestingly, although applying the offset was expected to improve the model’s statistical score, it resulted in lower statistical agreement across all four models (Table 3).

A comparison of each pair of models (both with and without an offset, with the same number of OxCal Phases) highlights the models’ sensitivity to offset assumptions and suggests that a fixed offset of 11 ± 5 BP may not be appropriate for this period. This is consistent with previous studies indicating that the magnitude of the offset may vary over time and cannot be represented by a single fixed value [28,33]. Unfortunately, there is currently insufficient data to indicate the magnitude of the offset during the Early Roman period. Accordingly, the results presented in this study are based on the model which provides the highest statistical agreement under the tested assumptions - ‘4-Phase’ model (without an offset), hereafter referred to as the ‘preferred model’.

### The ’preferred model’

The preferred model consists of four OxCal Phases. In the earliest OxCal Phase, we included two logs from a building of phase 1b that was covered by the main palace fort structure of Herodium. The second OxCal Phase comprises the construction of the Mountain Palace-Fortress and some of the identified alterations (phases 2-3b). It includes beams from two lintels from the construction phase of the palace-fortress (Phase 2a), an additional lintel from the later alterations phase (Phase 2b), and a wooden post from the artificial mount construction phase 3a-b. Multiple rings were sampled from the latter two. The third OxCal Phase in the model includes a single beam from construction phase 3c, which postdates the building of the artificial mound. The last OxCal Phase comes from the tunnel systems associated with the Bar Kokhba revolt and includes four logs associated with construction phase 5.

A comparison of the modeled date ranges with the calibrated dates without a chronological model is given in S1 Table. The unmodeled, calibrated dates are plotted in S1 Fig.

### Phase 1b: Prior to Herod's main construction activities

Only sparse remains are attributed to this phase, although the construction of the round “Eastern Tower” on the mountaintop might have been built already at this phase [4]. A massive rectangular structure, buried by the artificial filling of the volcano-like mound, was partially uncovered on the northeast slope, next to the wide stairways (structure A2063, Fig 3). The structure includes a series of horizontal wooden beams extending beyond the structure’s north wall. The rounded beams are 10–20 cm in diameter, and their length is unknown. Although only a small segment of the structure was exposed, it seems that the beams were probably integrated into the foundation of the building as shock absorbers or spacers.

**Fig 3:**
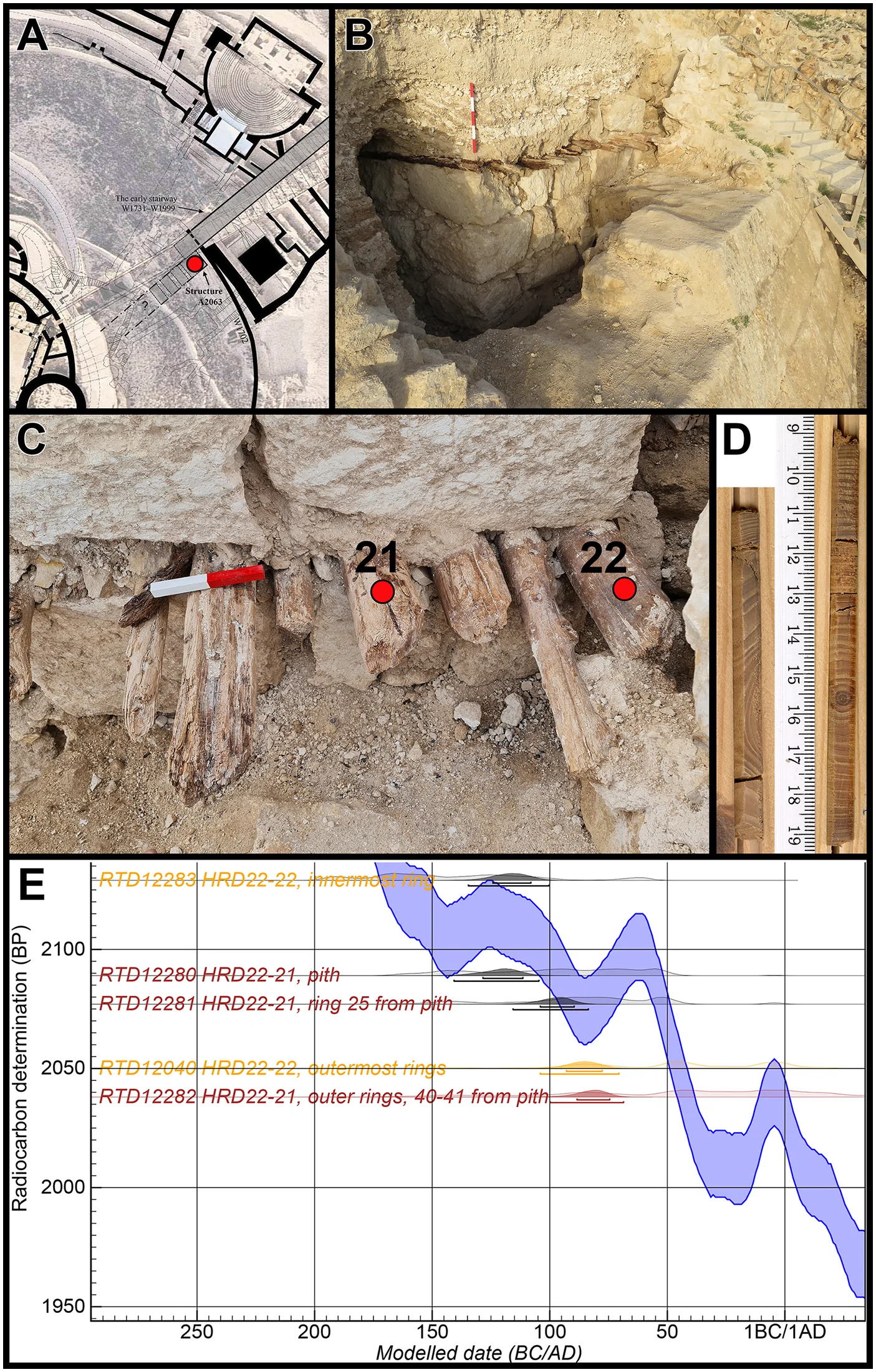
Phase 1, Samples HRD22−21 and HRD22−22. A. Location of the structure within the site (red circle). B. The excavated corner of the structure with the wooden beams foundation layer. C. The sampled beams (red circles). Scalebar is 20 cm. D. The polished wooden cores of HRD22−21 (left) and HRD22−22 (right) before sampling. E. A ‘curve plot’ of the ^14^C results of HRD22−21 (brown) and HRD22−22 (yellow). The outer rings of both fall at 90−70 BC.

The two best-preserved beams were sampled by coring (Fig 3C, red circles) and were dated by several rings each to allow wiggle matching; from log HRD22−21 sample **RTD 12280** was measured from the innermost ring, **RTD 12281** was taken from 25 rings later, **RTD 12282** from another 16 years later and the latest, outermost ring, **RTD 12039** about two rings later. However, **RTD 12039** was identified as an outlier (S2 Fig). From log HRD22−22, sample **RTD 12283** was taken from the innermost ring, while the outermost ring, about 31 growth rings later, was measured as **RTD 12040**. The felling date of both logs was modeled between 100−90 and 70 BC (68.3% probability) and 110 and 70−60 BC (95.4% probability).

### Phase 2a: Early Herod's 1st major construction activities

During this phase, the fortified palace on the mount was built, along with most of the structures on the northern slope, such as the theater and the mausoleum. This phase is dated to 30−15 BC based on historical and stratigraphic considerations [4,48]. The fortified palace's construction began with the building of the outer wall, with its three half-circular towers and the peripheral corridor. The corridor seems to have had five stories (only three survived) and two underground basement levels.

Two lintels were sampled from entrances to the basement floor below the main entrance room to the palace (room H40). From the southeast entrance, an outermost part was peeled off the beam HRD20−8 in the field and dated as **RTD 10489** (Fig 4). This may have been the outermost ring of the stem due to the shape and darker color of the sampled part. From the northwest entrance, log HRD20−9 was dated twice as **RTD 10490**, and the results were combined using the R_combine function (Fig 5). This log was partly decayed, and the outer part of the stem was not preserved (Fig 5). The felling dates of both logs are the beginning of the second half of the 1^st^ century BC, between 50−20 BC (68.3% probability) and 50−10 BC (95.4% probability), coinciding with or slightly preceding the construction date based on the historical sources derived date.

**Fig 4:**
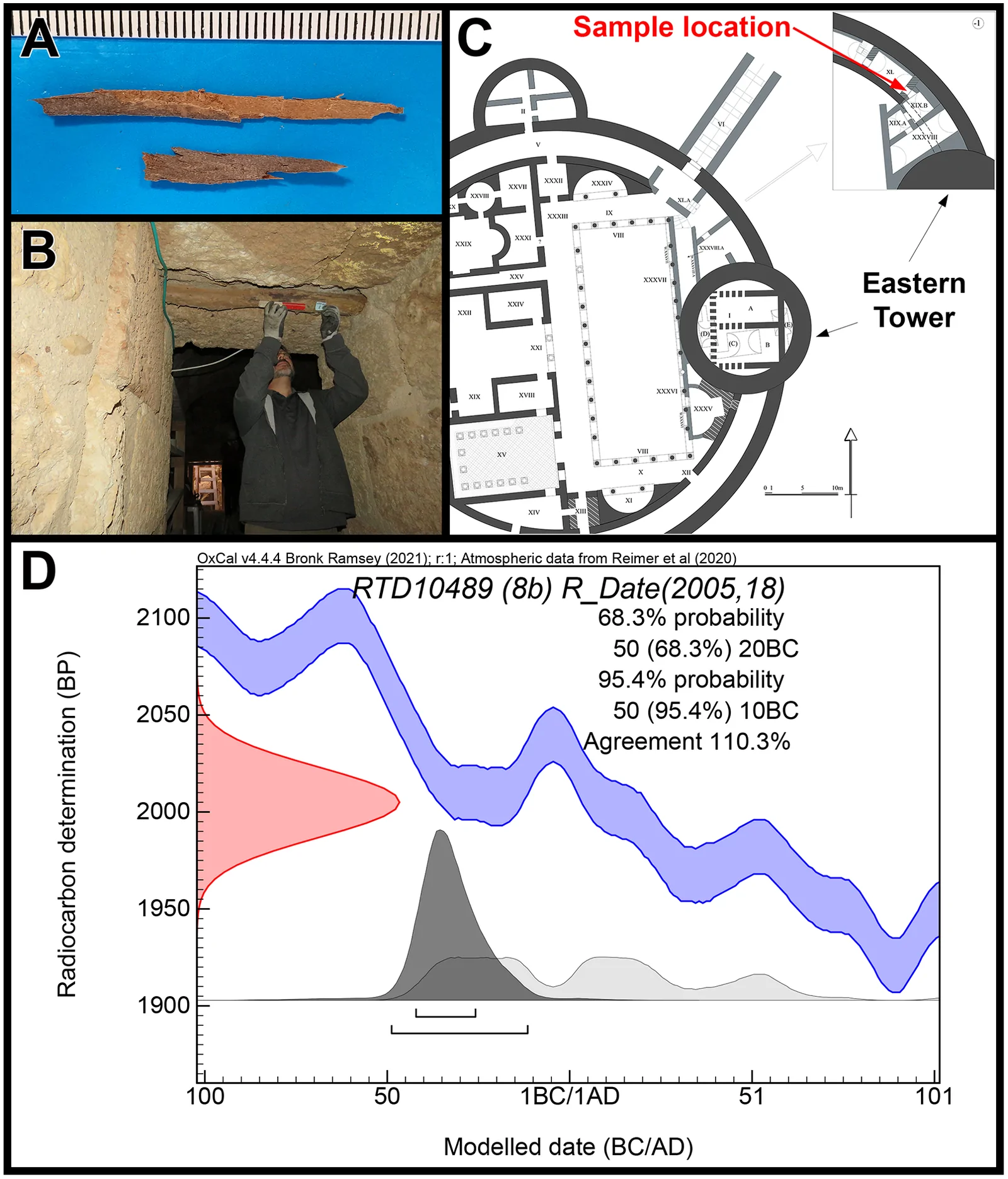
Sample HRD20-8b (RTD 10489). A. The peeled pieces from the outermost part of the beam. B. The lintel beam in situ. The sample was taken from the darker part above the 20 cm scale bar. C. The beam’s location (red arrow) in the palace plan, below the entrance room. The plan on the upper right is for the basement floor. D. The modeled dating result – 50−20 BC (68.3% probability).

**Fig 5:**
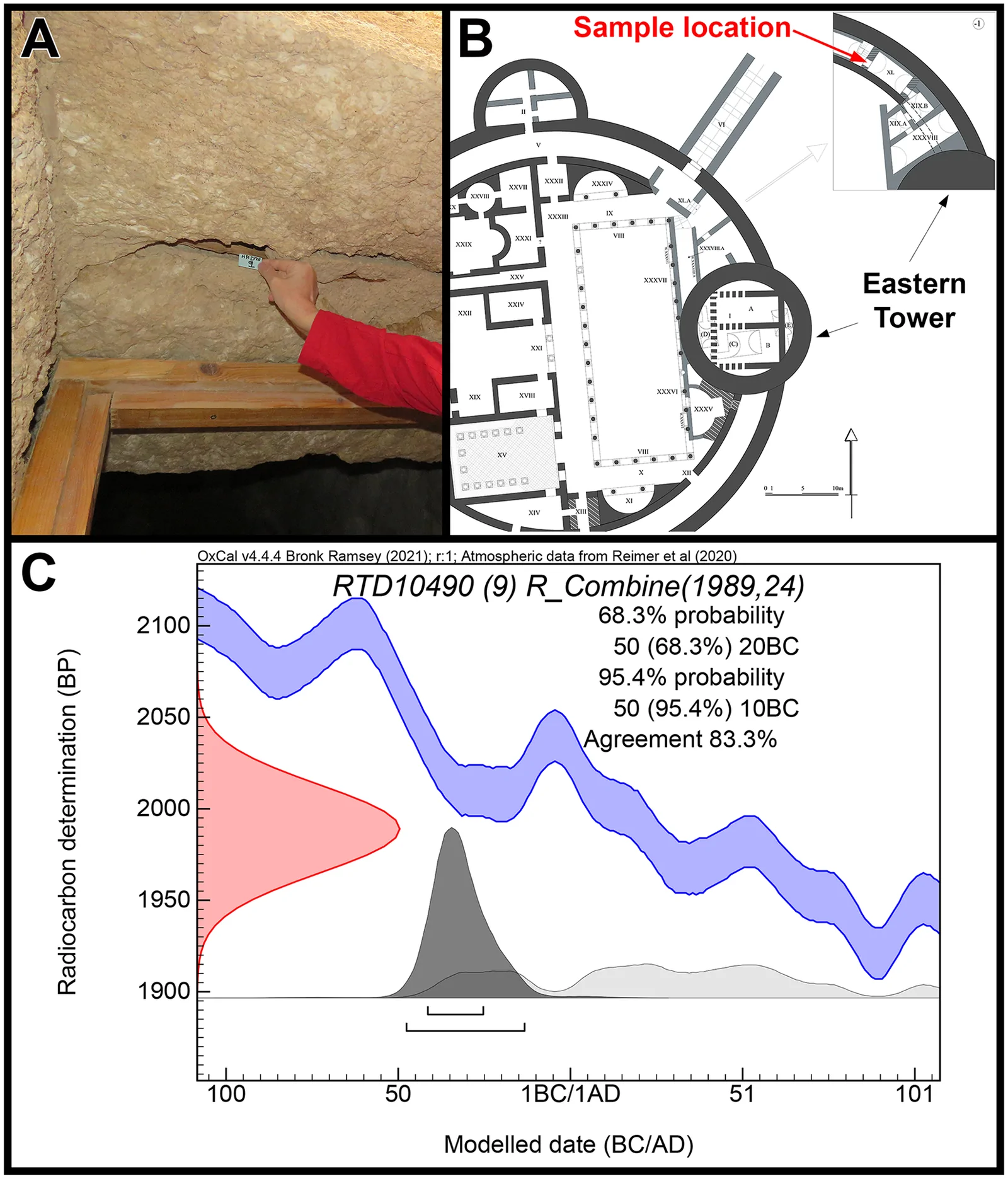
Sample HRD20−9 (RTD 10490). A. The eroded lintel beam in situ. B. The location of the sample on the palace plan, below the entrance room. C. The combined modeled dating result of the two measurements of this sample is 50−20 BC (68.3% probability).

### Phase 2b: Late Herod's architectural alterations

Several structural alterations were performed during the site's active years. The most altered location in this phase is the eastern wing of the mountain palace-fortress, to the south and north of the east round tower [4,48,49]. New internal room divisions were made on the ground floor and the basement. We sampled a well-preserved wooden lintel situated above one of the new doors leading from the entrance room basement towards the southeast. The lintel included four rectangular cypress beams, of which three survived. The middle beam was cored (HRD20-10A). The innermost ring was dated as **RTD 12284**, the 16^th^ ring after it was dated as **RTD 12285**, and 11 rings after that, two outermost rings were dated as **RTD 10643** (Fig 6). As for phase 2a, the beam from phase 2b was felled at a similar time range during the beginning of the second half of the 1^st^ century BC (the 68.3% probability falls between 50−30 BC, and the 95.4% between 60−20 BC).

**Fig 6:**
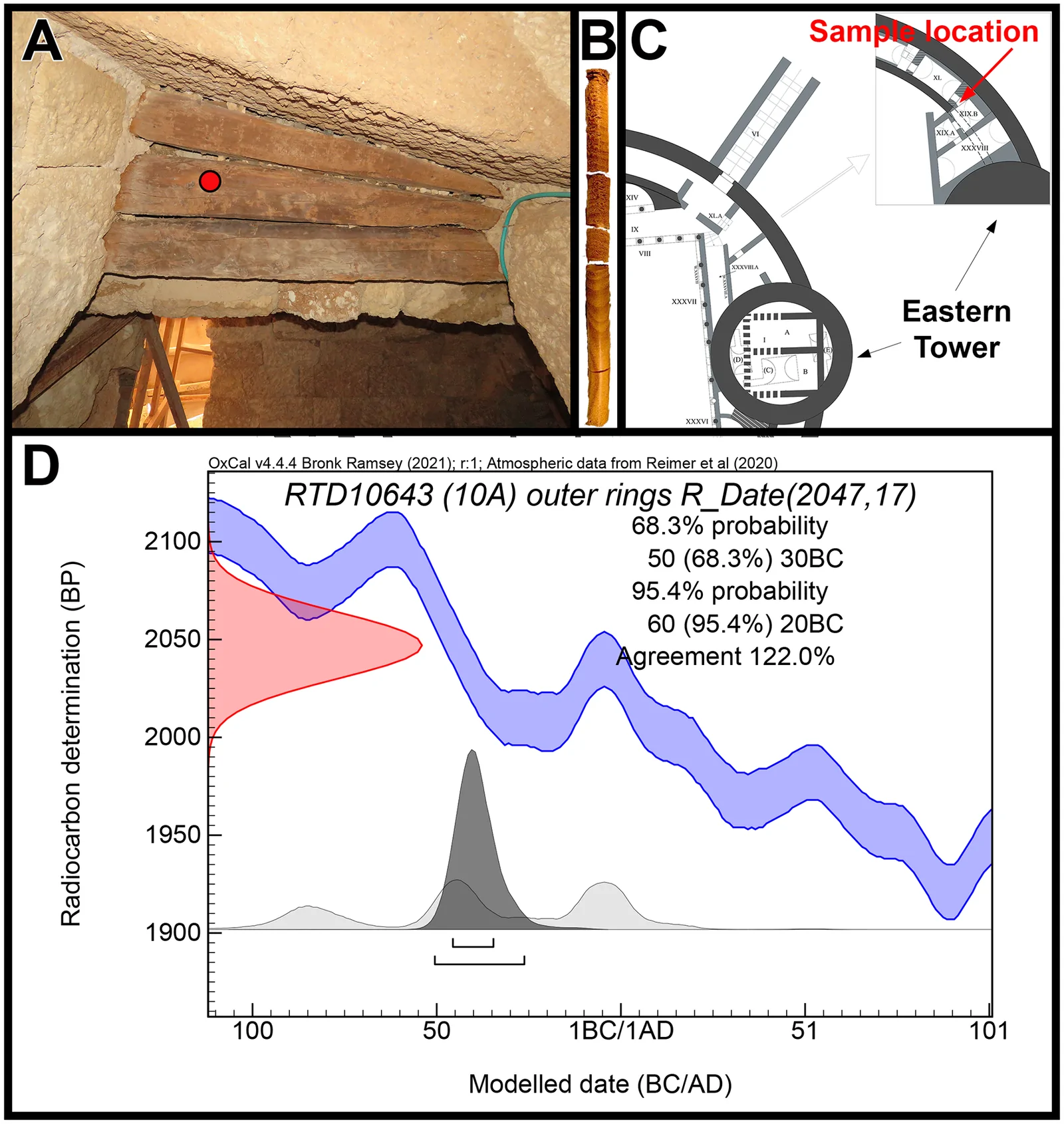
Sample HRD20-10A from the east wing modifications. A. The lintel beams in situ. The core’s location is marked by a red circle. B. The core itself (the width is 0.8 cm). The outermost ring was sampled for dating. C. The location of the wooden lintel in the palace plan, at the south-east entrance to the basement. D. The modeled dating result – 50−30 BC.

### Phase 3a-b: Late Herod's artificial mound construction

As part of transforming the mountain into Herod's tomb complex, the slopes outside the rounded fortress were ‘buried’ and covered with earth, forming a volcano-like artificial mound. At first, an impressive arched corridor was built to serve as the site's royal entrance. However, a later replanning canceled the passage and filled it with earth as part of a possible expansion of the artificial mound [4,48,50]. On top of the now-blocked corridor and against its edge, a steep staircase was built using a broad stone foundation. The foundation north of the canceled arched corridor was composed of two parallel walls, erected in several phases. Each phase included temporal supports of the walls, by stones, and probably also by placing wooden beams from one wall to the other, preventing them from collapsing inwards. One such wooden post, sample HRD20−13, was embedded within the fill of this later-stepped pathway (Fig 7). Thirty growth rings were counted in the field, from which 25 were identified in a sampled core (Fig 7D). The outermost rings are preserved mostly within the fill and were sampled separately in the field (Fig 7B, just below the blue tag). Growth rings 2, 18, and 29−30 were dated as **RTD 10666, 10667, and 10491**, respectively (Figs 2 and 7E). The modeled calibrated date of the outermost rings is 50−30 BC, within the second half of the 1^st^ century BC, similar to the felling dates from the previous two phases. The wood was identified as fir (*Abies alba*), a non-local species.

**Fig 7:**
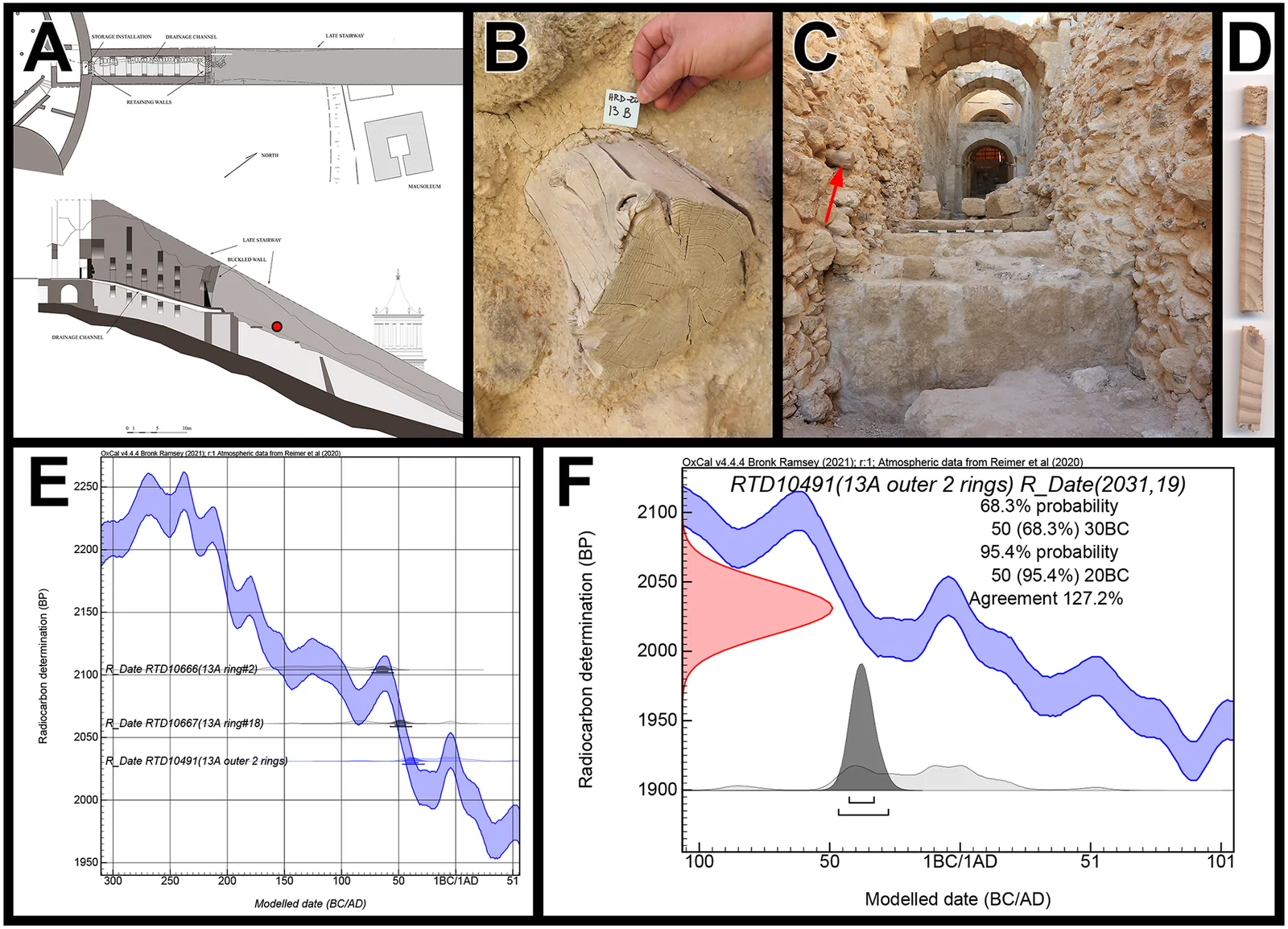
Sample HRD20-13A from the artificial mound construction. A. Location of the wooden post in the latest stepped pathway, marked by a red circle. B. The post itself, with its easily seen growth rings. Note that the outermost rings are mostly absent in the exposed part of the post but are present close to the wall (below the blue tag). C. A wider field image. The log is marked by a red arrow. D. The extracted core. 25 rings can be seen from the pith. The width is 0.8 cm. E. A ‘curve plot’ of the three dated samples of rings 2, 18, and 29−30, following perfectly a short slope in the calibration curve, which fixes the date to a narrow range. F. The modeled dating result of the outer rings – 50−30 BC (68.3% probability).

### Phase 3c: Late Herodian, post-artificial mound construction

After constructing the artificial mound, two short and narrow corridors were built, enabling access to the water reservoirs through the artificial fills. The corridors were strengthened by round wooden cypress beams, each around 2 meters long, from the corridor walls into the mound's fill [51]. One of the beams, HRD20−15, dated as **RTD 10492** (Fig 8), was taken from the entrance arch to the large water reservoir. It is a thinner post than the previous samples; therefore, only the outer rings were sampled in the field. The model places it between 30 BC and 10 AD. However, this sample can also statistically fit within OxCal Phase 2 (S3 Fig). In such a scenario, the felling date of **RTD 10492** is earlier, between 50 and 10 BC.

**Fig 8:**
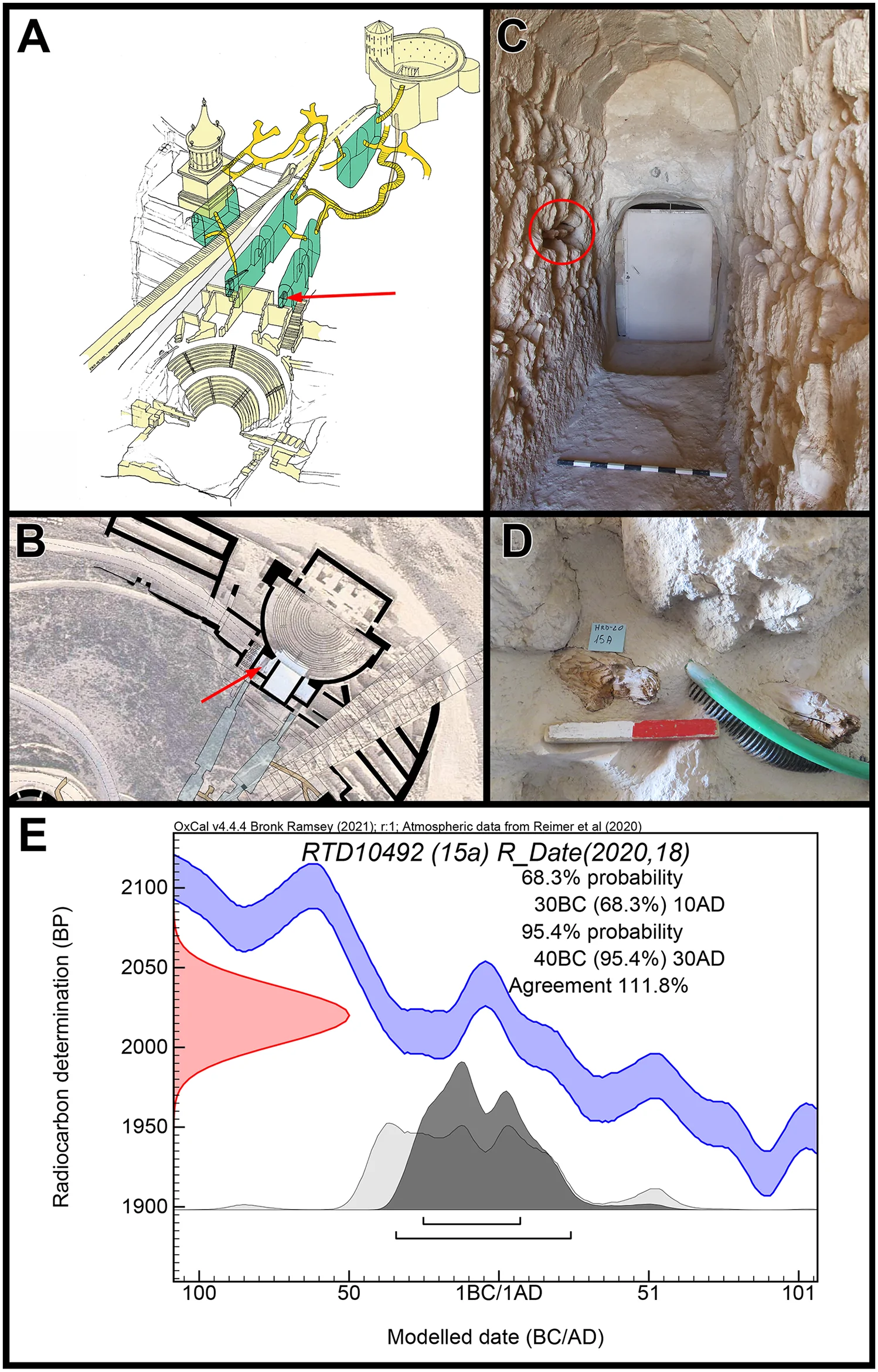
Sample HRD20-15A from the artificial mound construction. A-B. The location of the wooden post at the entrance arch to the large water reservoir. B. The post in situ, marked by a red circle. C. A zoom-in on the sample. The scale bar is 20 cm. The outermost ring was scraped from the top of the post, left to the tag. D. The modeled dating result – 30 BC – 10 AD (68.3% probability).

### Phase 5: Bar-Kokhba Revolt

The use of wood during the Bar Kokhba revolt was widespread. It was used as a building material in the living quarters and public facilities but mainly in the tunneling system that was dug throughout the mountain and fortress as a central part of the rebels’ military strategy. These tunnels were roofed by wooden beams, leaning on wooden poles on both sides of the tunnel [13,50]. One beam from such roofing was dated in a tunnel dug through the constructive fill at the base of the arched corridor (Fig 9). The beam, HRD20–18, was sawed and polished, and two single rings were sampled – the 3^rd^ innermost one, **RTD 11464**, and the outermost ring, **RTD 11465**, counted as the 15^th^. Even though this beam was identified as a cypress (*Cupressus sempervirens*), which is known to form false rings at times, as the beam contains no more than 15 rings, we consider the possible error in ring count negligible. The outer ring's modeled date ranges between 80–120 AD.

**Fig 9:**
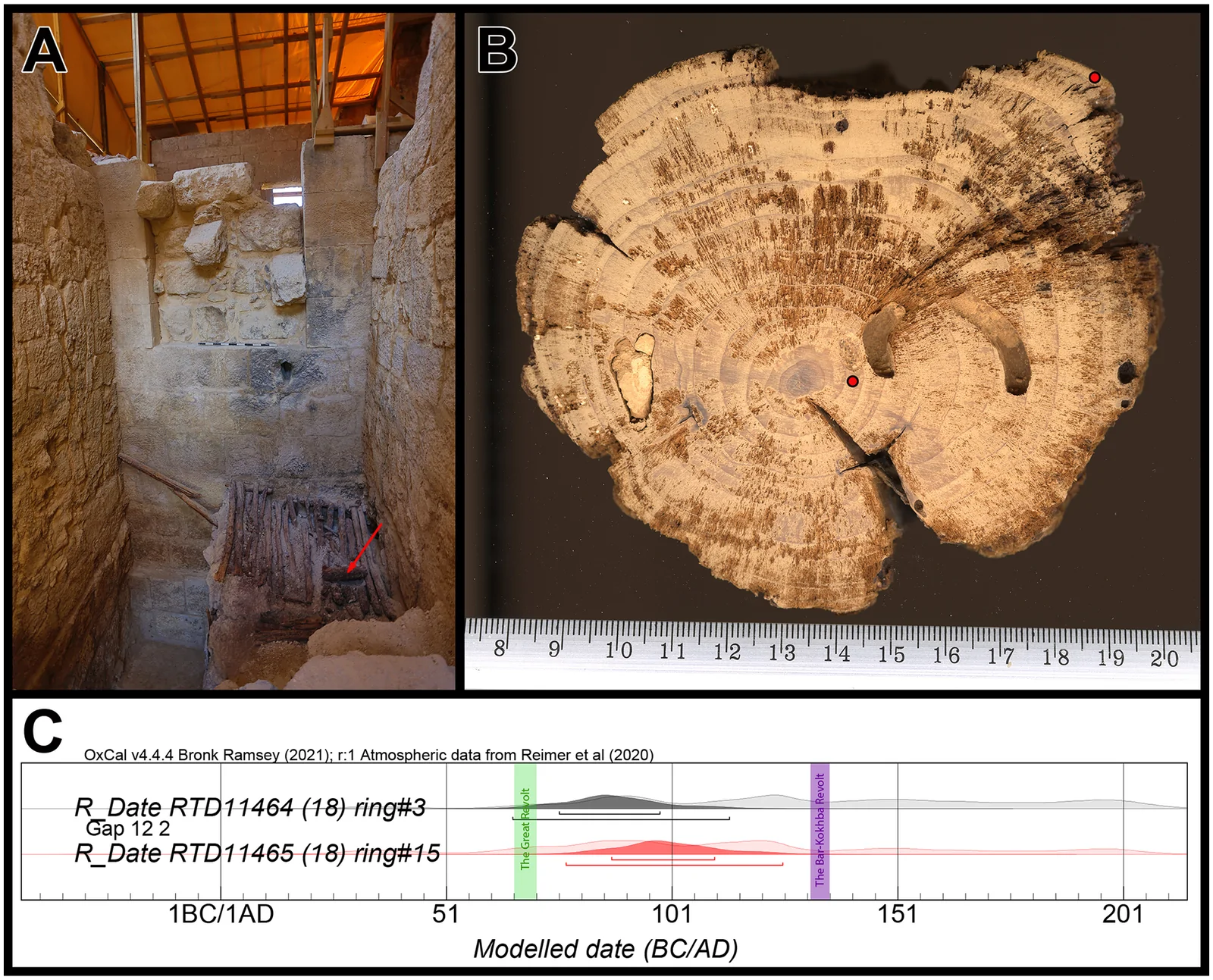
Sample HRD20-18 from a Bar-Kokhba tunnel. A. The sampled log (red arrow) in situ. B. A polished section of the log. Red circles mark sampling locations on the 3^rd^ and 15^th^ rings. C. A ‘multi plot’ view of the modeled dates. The green bar marks the time of the Great Revolt (66-71 AD), and the purple bar the 132-136 AD Bar-Kokhba revolt.

A second location from this phase was dated from a wooden ceiling of a tunnel built between the two basement floors at the fortress’ north wing peripheral corridor. Two logs were sampled (Fig 10, HRD19–4,5). The core extracted from HRD19–4 was only 6 cm long, with only a handful of visible rings. Therefore, the relative location of the dated ring **RTD 10304** could not be specified in the sampling process. The modeled date ranges between 70–110 AD (Table 2 and Fig 10C). HRD19–5 was a pine tree log consisting of 30 rings, from which the inner ring # 2 was dated as **RTD 10335.** The outermost ring was dated twice as **RTD 10305** and **10305.1**. The two measurements of that ring were combined using the R_combine function in OxCal. The modeled age of the outer rings is 90–120 AD (Table 2 and Fig 10C).

**Fig 10:**
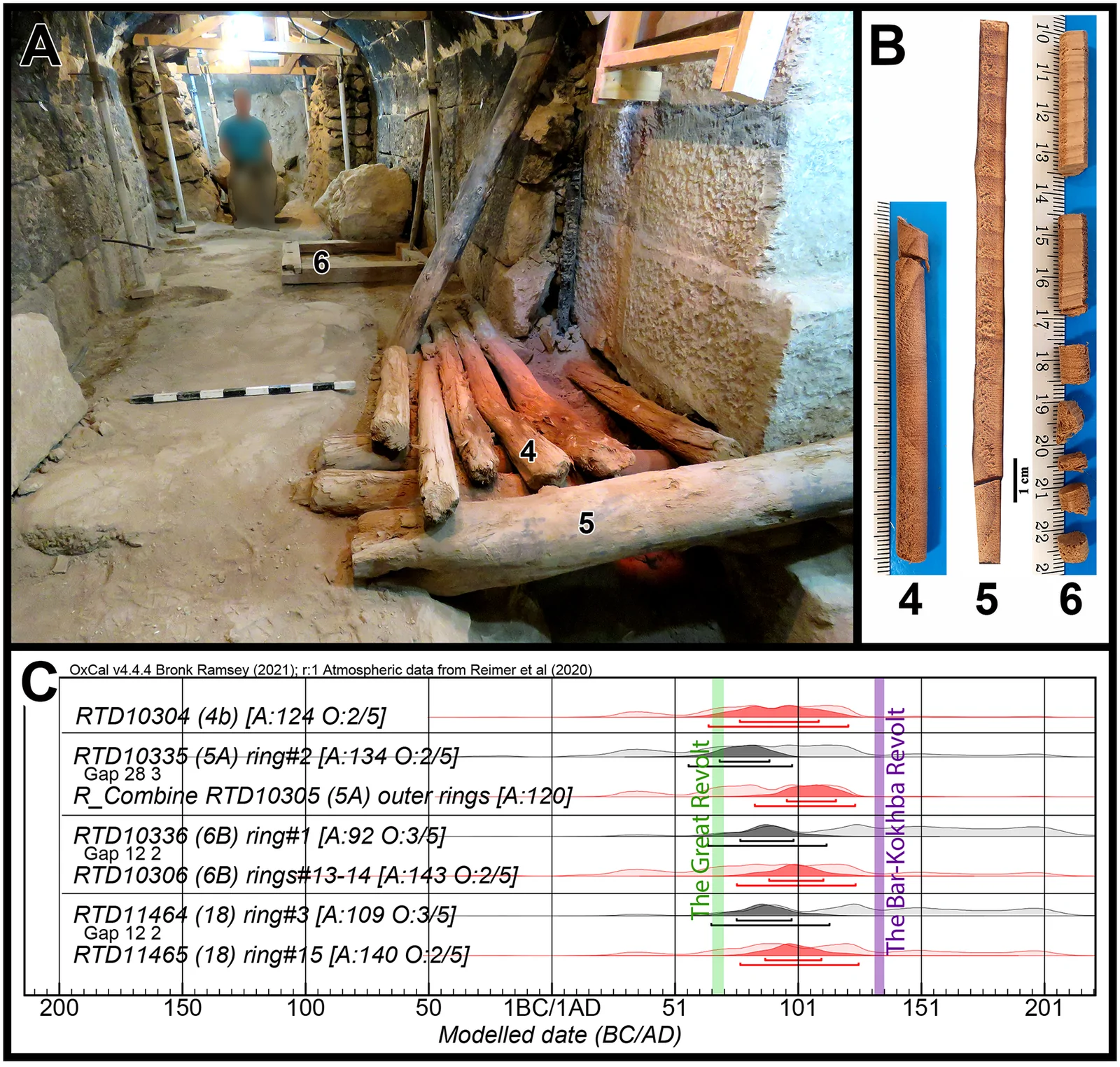
Samples HRD19-4,5,6 from a corridor just above one of the tunnels of Bar Kokhba. A. The logs in situ with the square wooden installation in the background. B. The cores of the three samples. C. A ‘multi plot’ view of the calibrated, modeled dates of the three samples. The red distributions are of the outer rings of each wood. The green bar marks the time of the Great Revolt (66-71 AD), and the purple bar the 132-136 AD Bar-Kokhba revolt.

A square wooden installation made of a cedar tree (*Cedrus libani*) and of unknown use was found several meters away (Fig 10, HRD19–6). Ring #1 (**RTD10336**) and rings #13–14 (**RTD10306**) were dated, both from the longest piece of the core, consisting of a total of 14 rings (Fig 10B, top part of sample 6). The modeled result of the outer rings is 80–120 AD (Table 2 and Fig 10C).

Overall, the felling dates of the four wood samples associated with the Bar-Kokhba revolt phase are contemporaneous and interestingly later than the Great Revolt in 70 AD and earlier than the Bar-Kokhba Revolt in 132–136 AD, raising the question of the origin of the tree logs.

## Discussion

The dating of the felling times of the beams used in the complex of Herodium has several implications for our understanding of the site’s history and its inhabitants, taking into consideration that the tree-felling times provide terminus post quem constraints on the associated construction activities. Therefore, the actual construction timing may differ due to factors such as wood storage, transport, or the reuse of beams. The earliest dated beams were used in the Phase 1b rectangular structure on the mid-slope of the mound (structure A2063), covered by the early major construction of Herod. Interestingly, the felling dates of the two logs sampled from the structure overlap to an early range between 90 and 70 BC. This date coincides with the tumultuous reign of the Hasmonean Alexander Jannaeus (103−76 BC). This, however, is puzzling since to date, after some 60 years of excavations at Herodium, no Hasmonean remains have ever been uncovered, while earlier, Hellenistic remains were revealed in a few locations under the Herodian structures [1]. The alternative conclusion is that the building is an early, pre-palace Herodian structure that uses a batch of felled trees from an unknown source that dates back some 40 years. No other chronologically meaningful material was found, as this building was not excavated to the floor and the foundations. The radiocarbon results of the beams are the first Hasmonean-related find identified at Herodium. Interestingly, in all other major building sites of Herod the Great, an earlier Hasmonaean phase has been identified or mentioned in the historical sources [52]. Herodium was considered an exception, but this first hint might point to the same pattern. However, at this point in the site's study, we do not think the logs’ felling dates are sufficient to conclude a Hasmonean presence.

Moreover, when considering fewer total felling events, the beams from this early structure can be dated to the early years of Herod’s reign (See 2- and 3-Felling events Models in S3, S4 Figs, accordingly). These alternative models are less likely than the most preferred model but are still statistically valid. Therefore, even assuming the logs were used immediately after the trees were cut down, the Phase 1 structure’s date as an early Herodian cannot be overruled.

The possible date of structure A2063 as Hasmonean may further provide a solution to the most difficult archaeological issue in the Herodium study – the issue of the Early Stairway (W1706-W1707). This straight and wide stairway that led to the top of the mountain is in the orientation of this nearby structure, but both differ in their direction from the entire Herodian architectural axes system in the Greater Herodium [48,50,53]. If future studies support a Hasmonean date for the structure, it is possible that the Early Stairway and structure A2063 were built together already in the Hasmonean period as part of an ancient ascent system to the top of the mountain.

Two lintels were sampled from the major building phase of the Palace Fort. Both are from the story below the main entrance room. In both cases, only the outer rings could be measured without a sequence for wiggle matching. Their calibrated modeled range is between 50 and 20 BC, placing the felling time of the trees during the early reign of King Herod, as expected by the historical sources.

The two timbers sampled from the following two phases (2b: east wing modifications and 3a-b: the construction of the artificial mound) have identical felling time ranges. Each of the timbers was sampled three times along its growth radius, enabling a wiggle-match of the dating results. Both samples are firmly placed on the calibration curve, where the earliest rings of the logs grew around 80−50 BC, and their felling date was around 50−30 BC. Hence, we conclude that the trees used for building phases 2-3b originate from one or several close-in-time felling events. It is very plausible that at the onset of Herod's large building projects, large shipments of wood were shipped from abroad (as the non-local fir, *Abies alba*, log found in Phase 3b), and together with locally collected wood, the timbers were stored, and the surplus was used later. Another scenario is that some of the wood was reused for later construction. Whatever the case, the timber for these phases was felled most likely before 30 BC.

The timber taken from the post-construction of the artificial mound (Phase 3c) stratigraphically postdated the timbers in Herod’s constructions. It was accordingly placed in the model later, giving it a felling date between 30 BC and 10 AD. This range is in good agreement with the historical sources, placing this phase around, or soon after, Herod’s death. Yet another plausible scenario is that this wooden post was part of the earlier felling event during Herod’s time. The 2-felling events model simulating this option finds it statistically valid (although with a relatively lower agreement value) with a modeled date range of 50 BC to 10 AD. Such a scenario points to the overcutting of wood at the beginning of the project, with organized facilities and manpower to maintain and oversee the storage for years. Unfortunately, the results regarding this possibility are not straightforward.

The latest sampled contexts turned out to be very informative for understanding the site. The four sampled timbers originated from activity during the Bar Kokhba revolt between 132–136 AD. However, the four trees had a felling date around 80–120 AD, in a time called “between the revolts,” when no significant building projects were detected to date at the site. Therefore, the question of the timber’s origin arises. It is known that Herod planted vast gardens in the lower and upper parts of Herodium, which included cypress and pines [54]. But, even if the trees originate from the gardens, someone cut them down 30 years or so before the arrival of the Bar Kokhba rebels at the site and imported the cedar beam of the installation at about the same time. Moreover, the sampled cypress and pine were 15–30 years old when felled. It means that either they grew wild from the original planted trees or that someone continued to maintain and plant trees in the gardens after Herod’s death.

The sparse historical sources and archaeological evidence of the period between the Great and Bar Kokhba revolts at the site teach of a possible garrison at the fortress and an administrative center in Lower Herodium [14], which may explain the finding of imported cedar wood and the growth of younger trees. It is striking that all four beams yielded highly similar felling time, although they were found in two different Bar-Kokhba tunnels and are of different species (2 cypress, 1 pine, and 1 cedar). This find has an important role in our understanding that this “dark period” in the site's history had a significant occupation, with higher timber use and building efforts, which made sufficient wood available for secondary uses during the Bar Kokhba Revolt.

## Conclusions

We have used Bayesian modeling of radiocarbon measurements from wooden elements associated with various construction phases at Herodium to estimate the most probable number of tree-felling events that supplied the site with wood. We tested models with 2, 3, 4, and 6 felling events, including the possibility of applying a seasonal offset. The four-felling-events model without an offset was found to be the most probable. As we dated tree-felling events, which provide terminus post quem constraints, there may be temporal gaps between felling and use in the construction activities at the site.

Two trees from the first felling event were used in the earliest identified building at the site (Phase 1b), with a 90–70 BC overlap in the felling time of the two beams. As any felling event serves as a terminus post quem, the construction itself occurred either in the final days of Alexander Jannaeus, or, perhaps, early in Herod’s days, using beams that were cut about 40 years earlier. The second felling event was during Herod the Great’s early years, probably during the first 10 years. These trees were used in Phases 2a-3b for the construction of the palace-fortress and the erection of the artificial mound. The third event goes with some alterations before, or soon after, the death of Herod (Phase 3c) and is dated between 30 BC – 10 AD. The fourth felling event was made by the site's inhabitants between the two revolts, around 80–120 AD, and included importing a cedar beam. Interestingly, four beams from that time were found in two different tunnels from the Bar Kokhba revolt, some decades later.

## Supporting information

S1 Fig Unmodeled, calibrated OxCal plot. (PDF)

S2 Fig Identifying an outlier. (PDF)

S3 Fig OxCal model of 2-felling events. (PDF)

S4 Fig OxCal model of 3-felling events. (PDF)

S5 Fig OxCal model of 6-felling events. (PDF)

S6 Fig OxCal model of 2-felling events, including an offset. (PDF)

S7 Fig OxCal model of 3-felling events, including an offset. (PDF)

S8 Fig OxCal model of 4-felling events, including an offset. (PDF)

S9 Fig OxCal model of 6-felling events, including an offset. (PDF)

S1 Table Modeled and unmodeled calibrated date ranges. (PDF)

S1 Text OxCal codes. (DOCX)
