# Supplementary material for: Radiocarbon dating of wooden beams at Herodium palace-fortress: Modeling tree-felling events and their implications on site chronology
Source: PLoS One. 2026 Oct 15;21(10):e0352580. doi: 10.1371/journal.pone.0352580 (PMC13671659; doi:10.1371/journal.pone.0352580)
Supplement: Supplementary file 1 [file pone.0352580.s001.pdf]

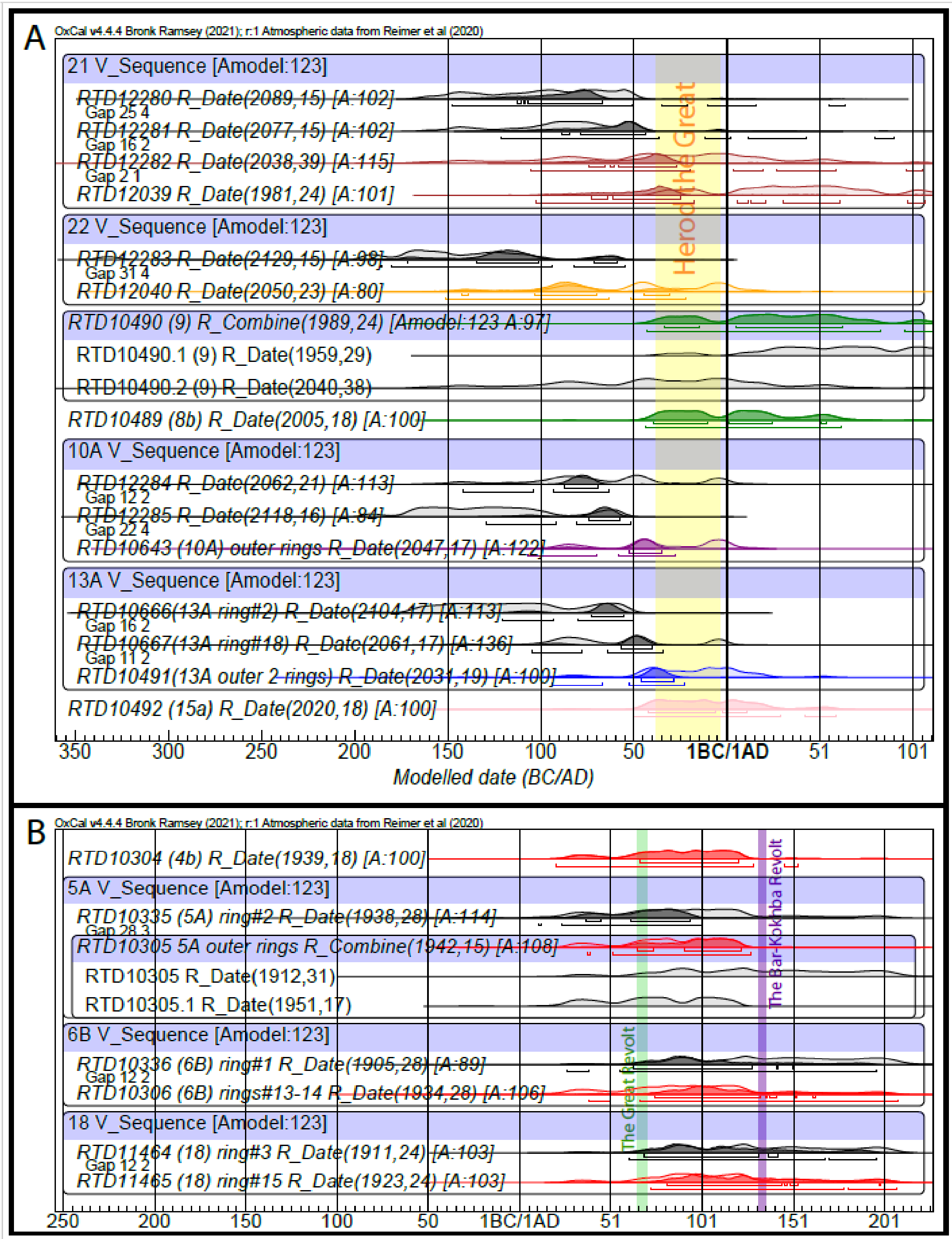

Figure S1. The unmodeled calibrated ranges. No stratigraphic data were included. Wherever several rings were dated from a single beam, V_Sequence was used, as in other models. The results were divided into A and B to increase the X-axis resolution. Note that the X-axis in A and B is different.
