# Supplementary material for: Radiocarbon dating of wooden beams at Herodium palace-fortress: Modeling tree-felling events and their implications on site chronology
Source: PLoS One. 2026 Oct 15;21(10):e0352580. doi: 10.1371/journal.pone.0352580 (PMC13671659; doi:10.1371/journal.pone.0352580)
Supplement: Supplementary file 2 [file pone.0352580.s002.pdf]

The identification of an outlier

First, we included all the samples in an identical model to the one given in Figure 2 without excluding any sample (Fig. S2). The actual difference in the model's code is that the Outlier probability of RTD12039 was set at 0.05 instead of 1, which defines it as a definite outlier.

In this model, the sample in red text (RTD12039) seems to be an outlier, as its statistical agreement in the model (marked by the value after the letter A) is much lower than the required 60%, and its probability being an outlier is way above the routine 5% probability (marked by the value after the letter O). The agreement of the whole model is 62, barely above the 60 threshold. Therefore, we defined it as a definite outlier and ran the model again. This time, the overall agreement increased to 158; all samples have an agreement above 60% and a probability of being an outlier of less than 5%. This model is our preferred model of the paper (Fig. 2).

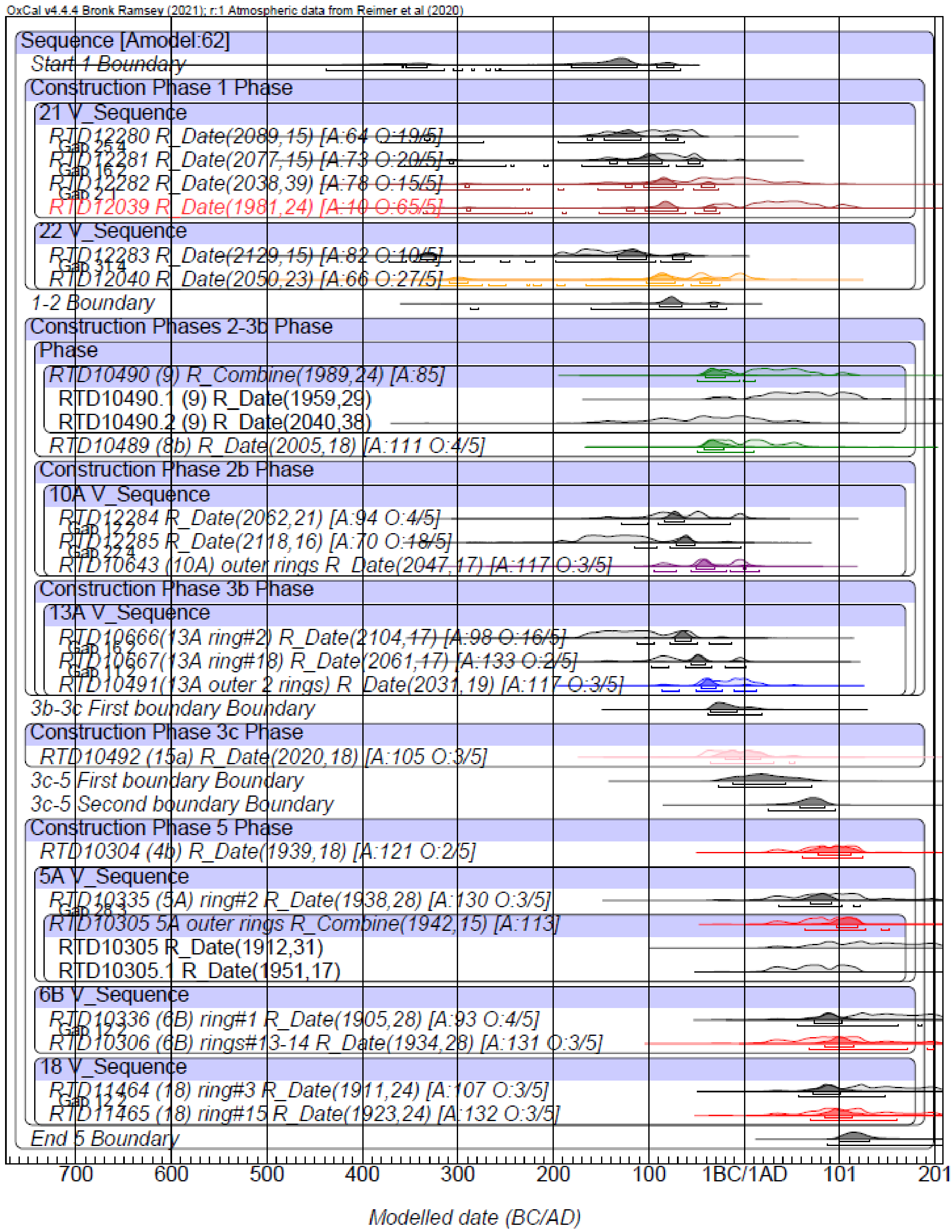

Figure S2. The preferred model, without defining any sample as a definite outlier. One sample has an agreement level below 60 (marked by red text).
