# Supplementary material for: Radiocarbon dating of wooden beams at Herodium palace-fortress: Modeling tree-felling events and their implications on site chronology
Source: PLoS One. 2026 Oct 15;21(10):e0352580. doi: 10.1371/journal.pone.0352580 (PMC13671659; doi:10.1371/journal.pone.0352580)
Supplement: Supplementary file 3 [file pone.0352580.s003.pdf]

2-felling events model

This model tests the statistical probability that the wood used for construction phases 1-3c was part of the same felling event, with a second event for phase 5. The model has a high statistical agreement value of 118, and no sample has an agreement below 60. However, 10 out of the 13 samples of Phases 1-3c (not including RTD12039) have an outlier probability higher than 5%. It means that although the model is statistically valid, most of the samples from these phases have a higher-than-normal chance of being outliers. These values point to the weakness of this model.

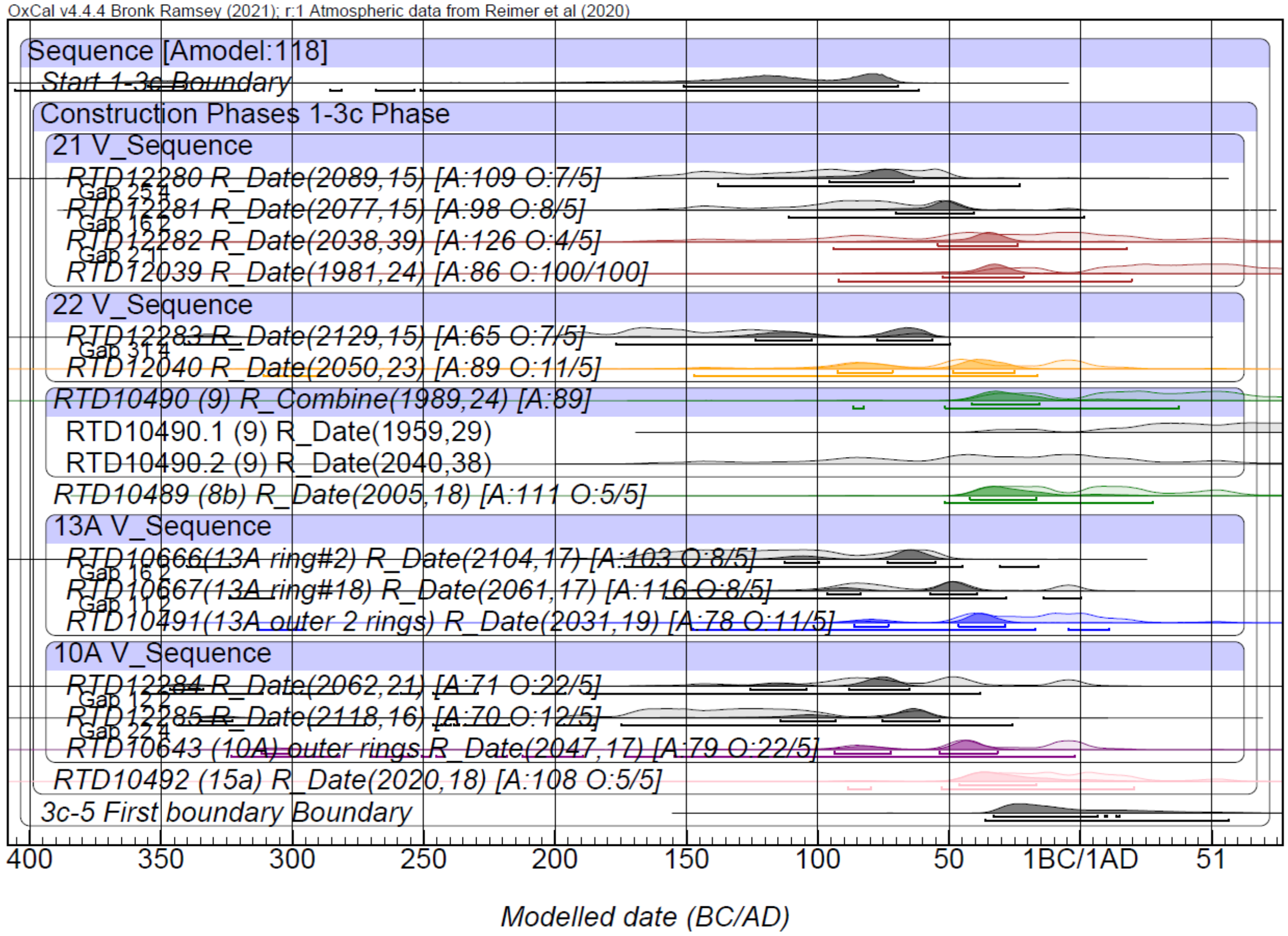

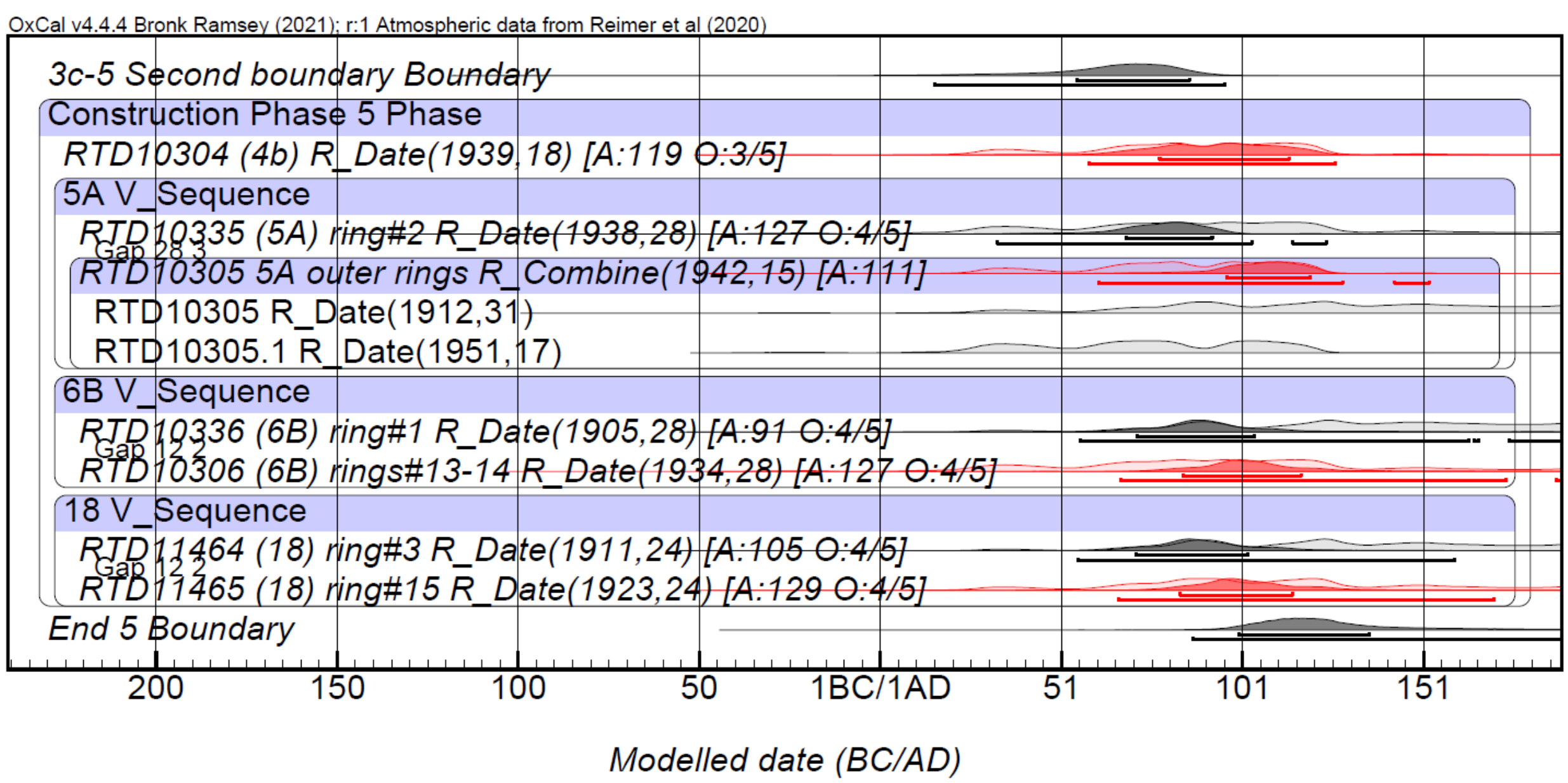

Figure S3. OxCal model with only two OxCal Phases, assuming all beams of Construction Phases 1-3c were part of the same felling event. The top figure shows the first OxCal Phase,

which includes the samples from Construction Phases 1-3c. The bottom figure shows the second OxCal Phase, which includes the samples from Construction Phase 5.
