# Supplementary material for: Radiocarbon dating of wooden beams at Herodium palace-fortress: Modeling tree-felling events and their implications on site chronology
Source: PLoS One. 2026 Oct 15;21(10):e0352580. doi: 10.1371/journal.pone.0352580 (PMC13671659; doi:10.1371/journal.pone.0352580)
Supplement: Supplementary file 4 [file pone.0352580.s004.pdf]

3-felling events model

In contrast to the 2-felling events model, here we exclude Construction Phase 3c and place it as a separate OxCal Phase. Hence, this model tests the statistical probability of a scenario that includes the following three felling events: First, for all of Herod's works (Phases 1-3b, assuming in this case that Phase 1 is an early Herodian phase). The second event is for Phase 3c (post-artificial mount construction), and the third is for Phase 5 (Fig S4).

Similar to the 2-felling events model, the 3-felling events model is also valid statistically, with a high overall agreement of 116. However, Sample RTD12284 has a low agreement of 59 (just below the required value of 60), and 9 out of the 13 samples of Phases 1-3c (not including RTD12039) have an outlier probability higher than 5%. It means that although the model is statistically valid, most of the samples from these phases have a higher-than-normal chance of being outliers. These values point to the weakness of this model as well, especially compared to the preferred model (Fig. 2), where none of the samples is above 5% outlier probability nor below the agreement of 60.

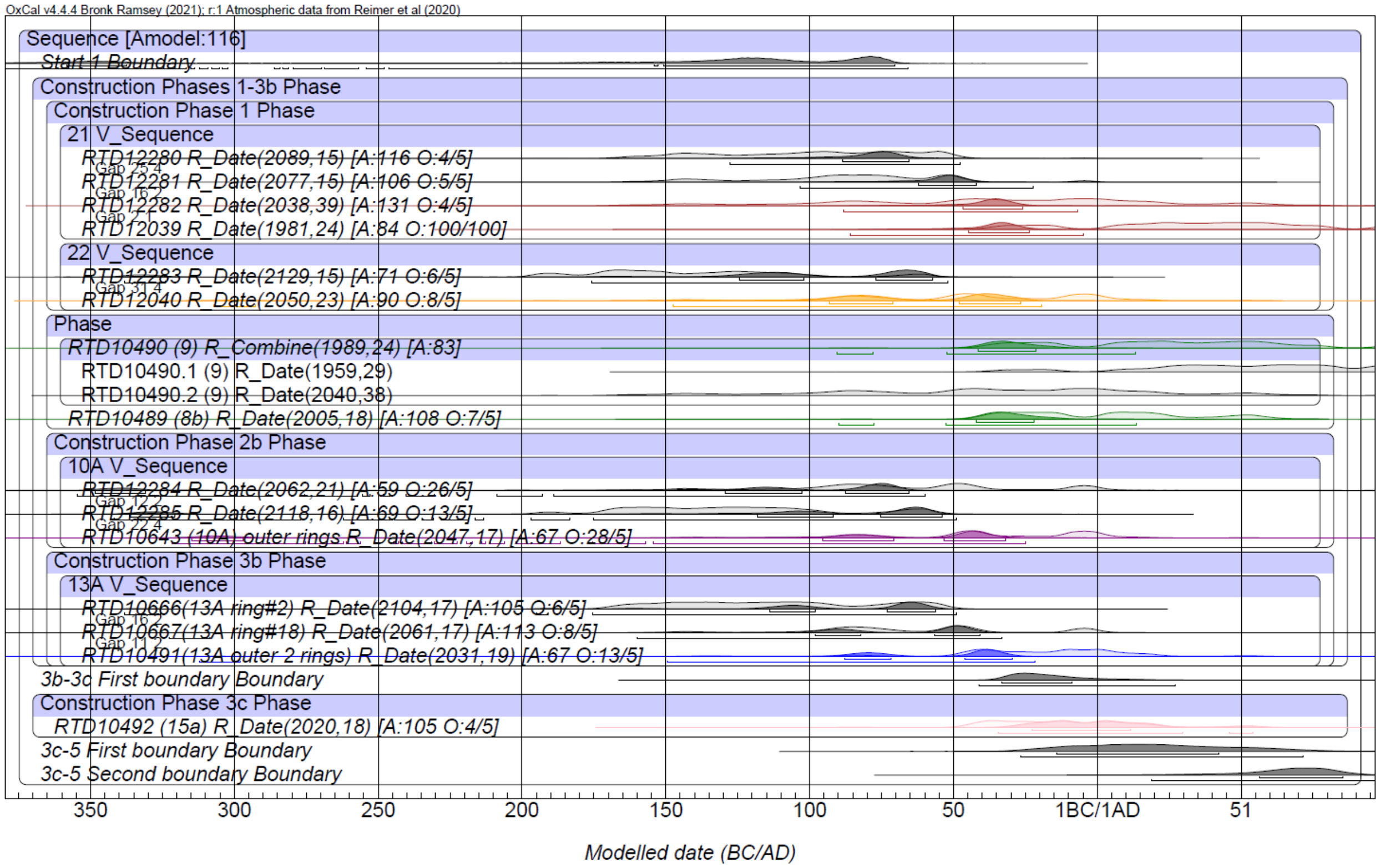

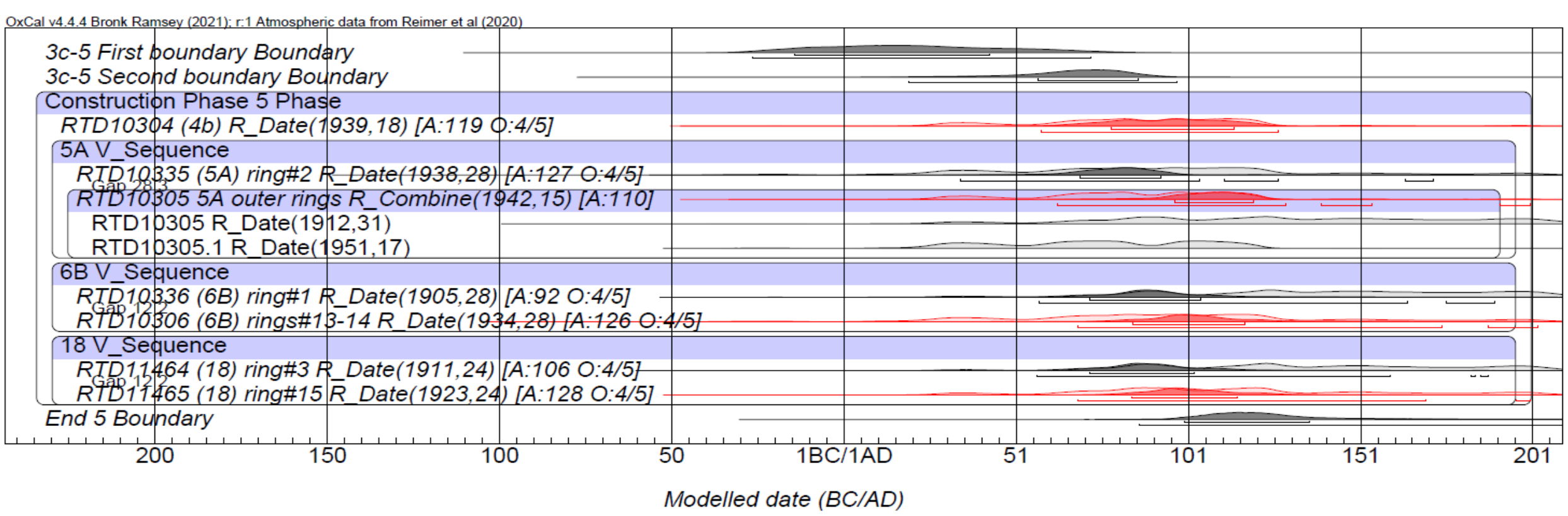

Figure S4. An OxCal model with three OxCal Phases, assuming all beams of Construction Phases 1-3b were part of the same felling events, Phase 3c had a separate felling event, and Phase 5 was the third one. This top figure shows the first two OxCal Phases, which include the samples from Construction Phases 1-3b and 3c. Phase 5 is in the bottom figure for an improved X-axis resolution.
