# Supplementary material for: Radiocarbon dating of wooden beams at Herodium palace-fortress: Modeling tree-felling events and their implications on site chronology
Source: PLoS One. 2026 Oct 15;21(10):e0352580. doi: 10.1371/journal.pone.0352580 (PMC13671659; doi:10.1371/journal.pone.0352580)
Supplement: Supplementary file 5 [file pone.0352580.s005.pdf]

6-felling events model

In this model, we tested the probability of the scenario in which each construction phase used fresh beams that were felled for that specific phase (S7 Fig. S7). The total agreement of this model is very low (Amodel: 12); three additional samples are identified as outliers, with two additional samples with an outlier probability slightly higher than 5. These parameters point to a low statistical probability of the validity of this scenario.

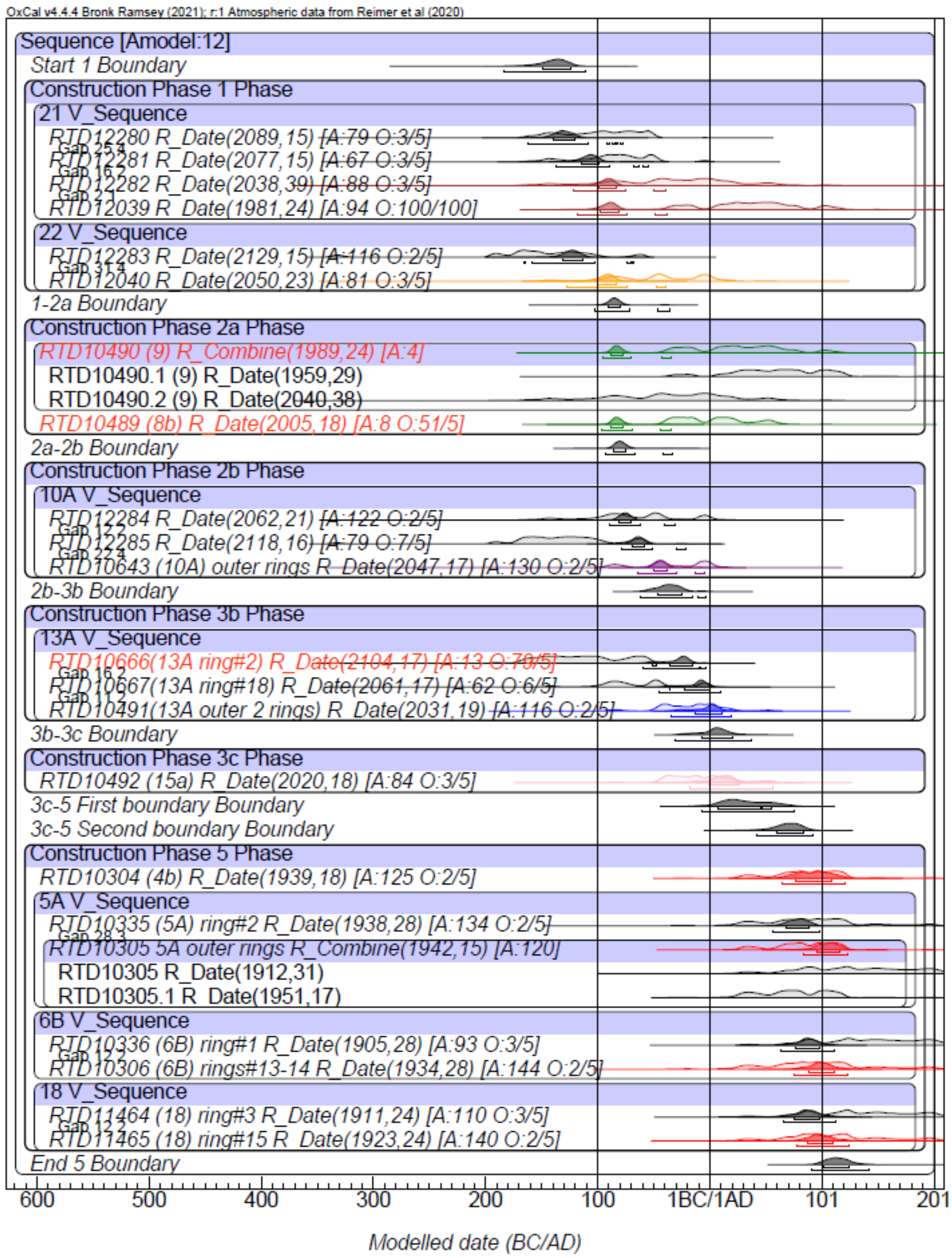

Figure S7. An OxCal model for the scenario in which each Construction Phase used new beams that were felled for that specific phase (six OxCal Phases).
