# Supplementary material for: Radiocarbon dating of wooden beams at Herodium palace-fortress: Modeling tree-felling events and their implications on site chronology
Source: PLoS One. 2026 Oct 15;21(10):e0352580. doi: 10.1371/journal.pone.0352580 (PMC13671659; doi:10.1371/journal.pone.0352580)
Supplement: Supplementary file 7 [file pone.0352580.s007.pdf]

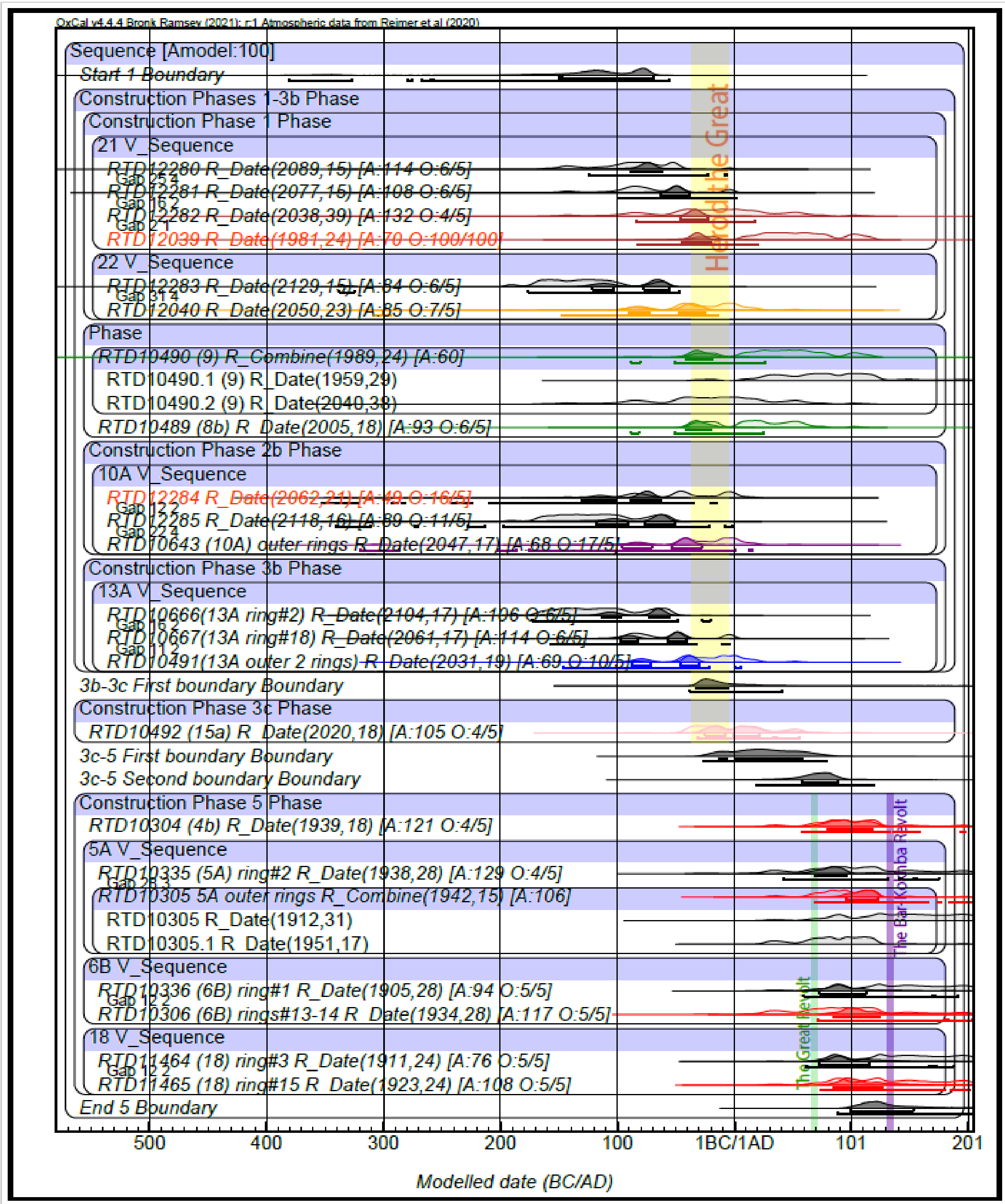

Figure S7. An OxCal model with three OxCal Phases, assuming all beams of Construction Phases 1-3b were part of the same felling events, Phase 3c had a separate felling event, and Phase 5 was the third one. An 11±5 seasonal offset was applied to cypress and pine samples, as well as to HRD20-9. Compare with Fig. S4, which does not include an offset.
