# Supplementary material for: Radiocarbon dating of wooden beams at Herodium palace-fortress: Modeling tree-felling events and their implications on site chronology
Source: PLoS One. 2026 Oct 15;21(10):e0352580. doi: 10.1371/journal.pone.0352580 (PMC13671659; doi:10.1371/journal.pone.0352580)
Supplement: Supplementary file 8 [file pone.0352580.s008.pdf]

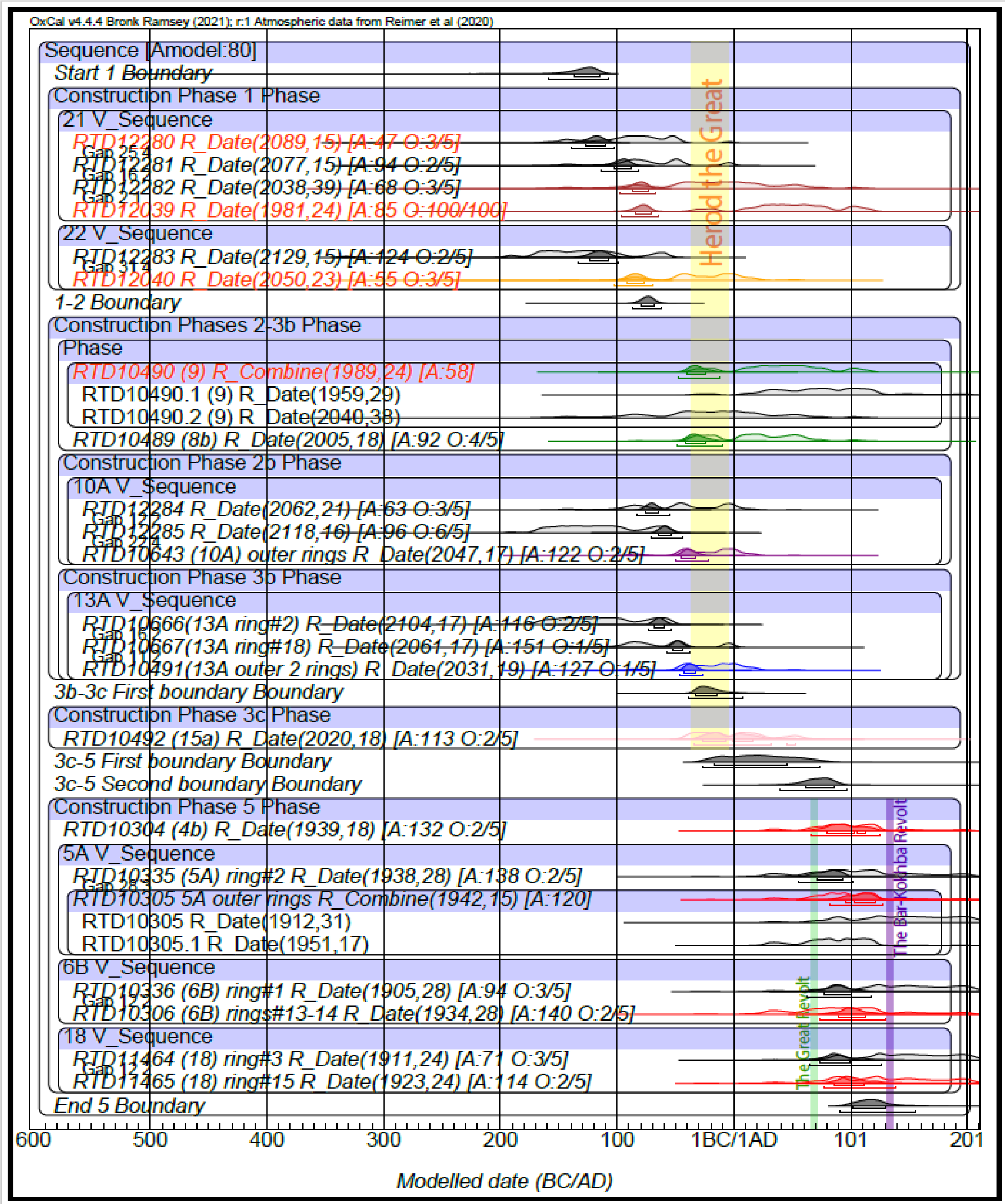

Figure S8. An OxCal model with four OxCal Phases, like the ‘preferred model’ (Fig 2). Here, an 11±5 seasonal offset was applied to cypress and pine samples, as well as to HRD20-9.
