# Supplementary material for: Radiocarbon dating of wooden beams at Herodium palace-fortress: Modeling tree-felling events and their implications on site chronology
Source: PLoS One. 2026 Oct 15;21(10):e0352580. doi: 10.1371/journal.pone.0352580 (PMC13671659; doi:10.1371/journal.pone.0352580)
Supplement: Supplementary file 10 [file pone.0352580.s010.pdf]

Table S1. A comparison between the modeled calibrated dates of the preferred model (Fig. 2 and Table 2) and the unmodeled calibrated dates (Fig. S1). No stratigraphic data were included. Wherever several rings were dated from a single beam, V_Sequence was used, as in other models.

**Sample No.**	**Modeled (68.3%)**	**Modeled (95.4%)**	**Unmodeled (68.3%)**	**Unmodeled (95.4%)**
RTD 12280	130BC(68.3%)110BC	150BC(95.4%)100BC	110BC (68.3%) 60BC	130BC (94.2%) 20BC<br>10BC (1.2%) 10AD
RTD 12281	110BC (68.3%) 90BC	120BC (95.4%) 80BC	100BC (68.3%) 40BC	100BC (92.9%) 20BC<br>10BC (2.3%) 30AD<br>80 (0.3%) 90AD
**RTD 12282**	**90BC (68.3%) 70BC**	**110BC (95.4%) 60BC**	**80BC (68.3%) 20BC**	**80BC (93.0%) 1BC<br>1AD (1.2%) 20AD<br>30AD (0.9%) 50AD<br>90AD (0.4%) 110AD**
RTD 12039*	Outlier. It was not part of the model.			
RTD 12283	130BC(68.3%)100BC	140BC(95.4%)100BC	180BC (3.2%) 160BC<br>140BC (57.9%) 100BC<br>80BC (7.2%) 60BC	190BC (95.4%) 50BC
**RTD 12040**	**100BC (64.2%) 70BC**	**110BC (95.4%) 70BC**	**150BC (3.7%) 130BC<br>110BC (58.5%) 70BC<br>50BC (6.0%) 30BC**	**160BC (95.4%) 20BC**
**RTD 10489**	**50BC (68.2%) 20AD**	**50BC (95.4%) 10BC**	**40BC (36.8%) 10BC<br>1AD (29.3%) 30AD<br>50AD (2.1%) 60AD**	**50BC (95.4%) 70AD**
**RTD 10490.1**<br>**RTD 10490.2**	**R_Combine<br>50BC (68.2%) 20BC**	**R_Combine<br>50BC (95.4%) 10BC**	**R_Combine<br>40BC (15.4%) 10BC<br>1AD (52.9%) 70AD**	**R_Combine<br>50BC (95.4%) 120AD**
RTD 12284	80BC (68.3%) 60BC	90BC (95.4%) 50BC	90BC (68.3%) 60BC	150BC (95.4%) 60BC
RTD 12285	70BC (68.3%) 50BC	80BC (95.4%) 40BC	80BC (68.3%) 50BC	140BC (95.4%) 50BC
RTD 10643	**50BC (68.3%) 30BC**	**60BC (95.4%) 20BC**	**60BC (68.3%) 30BC**	**120BC (95.4%) 20BC**
RTD 10666	70BC (68.3%) 50BC	80BC (95.4%) 50BC	80BC (68.3%) 50BC	130BC (95.4%) 50BC
RTD 10667	60BC (68.3%) 40BC	60BC (95.4%) 30BC	60BC (68.3%) 40BC	110BC (95.4%) 30BC
**RTD 10491**	**50BC (68.3%) 30BC**	**50BC (95.4%) 20BC**	**50BC (68.3%) 20BC**	**100BC (95.4%) 20BC**
**RTD 10492**	**30BC (68.3%) 10AD**	**40BC (95.4%) 30AD**	**50BC (68.3%) 20AD**	**60BC (90.4%) 30AD<br>40AD (5.0%) 60AD**
**RTD 10304**	**70AD (68.3%) 110AD**	**60AD (95.4%) 130AD**	**60AD (68.3%) 130AD**	**20AD (94.5%) 130AD<br>140AD (0.7%) 160AD<br>190AD (0.2%) 200AD**
RTD 10335	60AD (68.3%) 90AD	50AD (95.4%) 100AD	30AD (7.9%) 50AD<br>60AD (60.4%) 100AD	20AD (95.4%) 110AD
**RTD 10305.1**<br>**RTD 10305**	**R_Combine<br>90AD (68.3%) 120AD**	**R_Combine<br>80AD (95.4%) 130AD**	**R_Combine<br>60AD (9.0%) 80AD<br>90AD (59.3%) 130AD**	**R_Combine<br>50AD (95.4%) 130AD**
**RTD 10306**	**80AD (68.3%) 120AD**	**70AD (95.4%) 130AD**	**70AD (68.3%) 160AD**	**60AD (95.4%) 210AD**
RTD 10336	70AD (68.3%) 100AD	60AD (95.4%) 120AD	60AD (68.3%) 150AD	50AD (95.4%) 200AD
RTD 11464	70AD (68.3%) 100AD	60AD (95.4%) 120AD	70AD (68.3%) 150AD	60AD (95.4%) 200AD
**RTD 11465**	**80AD (68.3%) 120AD**	**70AD (95.4%) 130AD**	**80AD (68.3%) 160AD**	**70AD (95.4%) 210AD**
