# Supplementary material for: Radiocarbon dating of wooden beams at Herodium palace-fortress: Modeling tree-felling events and their implications on site chronology
Source: PLoS One. 2026 Oct 15;21(10):e0352580. doi: 10.1371/journal.pone.0352580 (PMC13671659; doi:10.1371/journal.pone.0352580)
Supplement: Supplementary file 11 [file pone.0352580.s011.docx]

*OxCal Models*

**The preferred model (4-Phases) used in the main manuscript**

Options()

{

Resolution=1;

};

Plot()

{

Outlier_Model("General",T(5),U(0,4),"t");

Sequence()

{

Boundary("Start 1");

Phase("Construction Phase 1")

{

V_Sequence("21")

{

R_Date("RTD12280",2089,15)

{

Outlier("General",0.05);

};

Gap(25,4);

R_Date("RTD12281",2077,15)

{

Outlier("General",0.05);

};

Gap(16,2);

R_Date("RTD12282",2038,39)

{

Outlier("General",0.05);

color="brown";

};

Gap(2,1);

R_Date("RTD12039",1981,24)

{

color="brown";

Outlier("General",1);

};

};

V_Sequence("22")

{

R_Date("RTD12283",2129,15)

{

Outlier("General",0.05);

};

Gap(31,4);

R_Date("RTD12040",2050,23)

{

color="orange";

Outlier("General",0.05);

};

};

};

Boundary("1-2");

Phase("Construction Phases 2-3b")

{

Phase(""Construction Phase 2a"")

{

R_Combine("RTD10490 (9)")

{

color="Green";

R_Date("RTD10490.1 (9)", 1959, 29)

{

Outlier("General",0.05);

};

R_Date("RTD10490.2 (9)", 2040, 38)

{

Outlier("General",0.05);

};

};

R_Date("RTD10489 (8b)", 2005, 18)

{

color="Green";

Outlier("General",0.05);

};

};

Phase("Construction Phase 2b")

{

V_Sequence("10A")

{

R_Date("RTD12284",2062,21)

{

Outlier("General",0.05);

};

Gap(12,2);

R_Date("RTD12285",2118,16)

{

Outlier("General",0.05);

};

Gap(22,4);

R_Date("RTD10643 (10A) outer rings", 2047, 17)

{

color="purple";

Outlier("General",0.05);

};

};

};

Phase("Construction Phase 3b")

{

V_Sequence("13A")

{

R_Date("RTD10666(13A ring#2)",2104,17)

{

Outlier("General",0.05);

};

Gap(16,2);

R_Date("RTD10667(13A ring#18)",2061,17)

{

Outlier("General",0.05);

};

Gap(11,2);

R_Date("RTD10491(13A outer 2 rings)",2031,19)

{

Outlier("General",0.05);

color="Blue";

};

};

};

};

Boundary("3b-3c First boundary");

Phase("Construction Phase 3c")

{

R_Date("RTD10492 (15a)", 2020, 18)

{

color="pink";

Outlier("General",0.05);

};

};

Boundary("3c-5 First boundary");

Boundary("3c-5 Second boundary");

Phase("Construction Phase 5")

{

R_Date("RTD10304 (4b)", 1939, 18)

{

color="Red";

Outlier("General",0.05);

};

V_Sequence("5A")

{

R_Date("RTD10335 (5A) ring#2",1938,28)

{

Outlier("General",0.05);

};

Gap(28,3);

R_Combine("RTD10305 5A outer rings")

{

R_Date("RTD10305",1912,31)

{

Outlier("General",0.05);

};

R_Date("RTD10305.1", 1951, 17)

{

Outlier("General",0.05);

};

color="Red";

};

};

V_Sequence("6B")

{

R_Date("RTD10336 (6B) ring#1",1905,28)

{

Outlier("General",0.05);

};

Gap(12,2);

R_Date("RTD10306 (6B) rings#13-14",1934,28)

{

Outlier("General",0.05);

color="Red";

};

};

V_Sequence("18")

{

R_Date("RTD11464 (18) ring#3",1911,24)

{

Outlier("General",0.05);

};

Gap(12,2);

R_Date("RTD11465 (18) ring#15",1923,24)

{

Outlier("General",0.05);

color="red";

};

};

};

Boundary("End 5");

};

};

**2-Phases Model**

Options()

{

Resolution=1;

};

Plot()

{

Outlier_Model("General",T(5),U(0,4),"t");

Sequence()

{

Boundary("Start 1-3c");

Phase("Construction Phases 1-3c")

{

V_Sequence("21")

{

R_Date("RTD12280",2089,15)

{

Outlier(0.05);

};

Gap(25,4);

R_Date("RTD12281",2077,15)

{

Outlier(0.05);

};

Gap(16,2);

R_Date("RTD12282",2038,39)

{

color="brown";

Outlier(0.05);

};

Gap(2,1);

R_Date("RTD12039",1981,24)

{

color="brown";

Outlier(1);

};

};

V_Sequence("22")

{

R_Date("RTD12283",2129,15)

{

Outlier(0.05);

};

Gap(31,4);

R_Date("RTD12040",2050,23)

{

color="orange";

Outlier(0.05);

};

};

R_Combine("RTD10490 (9)")

{

color="Green";

R_Date("RTD10490.1 (9)", 1959, 29)

{

Outlier(0.05);

};

R_Date("RTD10490.2 (9)", 2040, 38)

{

Outlier(0.05);

};

};

R_Date("RTD10489 (8b)", 2005, 18)

{

color="Green";

Outlier(0.05);

};

V_Sequence("13A")

{

R_Date("RTD10666(13A ring#2)",2104,17)

{

Outlier(0.05);

};

Gap(16,2);

R_Date("RTD10667(13A ring#18)",2061,17)

{

Outlier(0.05);

};

Gap(11,2);

R_Date("RTD10491(13A outer 2 rings)",2031,19)

{

Outlier(0.05);

color="Blue";

};

};

V_Sequence("10A")

{

R_Date("RTD12284",2062,21)

{

Outlier(0.05);

};

Gap(12,2);

R_Date("RTD12285",2118,16)

{

Outlier(0.05);

};

Gap(22,4);

R_Date("RTD10643 (10A) outer rings", 2047, 17)

{

color="purple";

Outlier(0.05);

};

};

R_Date("RTD10492 (15a)", 2020, 18)

{

color="pink";

Outlier(0.05);

};

};

Boundary("3c-5 First boundary");

Boundary("3c-5 Second boundary");

Phase("Construction Phase 5")

{

R_Date("RTD10304 (4b)", 1939, 18)

{

color="Red";

Outlier(0.05);

};

V_Sequence("5A")

{

R_Date("RTD10335 (5A) ring#2",1938,28)

{

Outlier(0.05);

};

Gap(28,3);

R_Combine("RTD10305 5A outer rings")

{

R_Date("RTD10305",1912,31)

{

Outlier(0.05);

};

R_Date("RTD10305.1", 1951, 17)

{

Outlier(0.05);

};

color="Red";

};

};

V_Sequence("6B")

{

R_Date("RTD10336 (6B) ring#1",1905,28)

{

Outlier(0.05);

};

Gap(12,2);

R_Date("RTD10306 (6B) rings#13-14",1934,28)

{

Outlier(0.05);

color="Red";

};

};

V_Sequence("18")

{

R_Date("RTD11464 (18) ring#3",1911,24)

{

Outlier(0.05);

};

Gap(12,2);

R_Date("RTD11465 (18) ring#15",1923,24)

{

Outlier(0.05);

color="red";

};

};

};

Boundary("End 5");

};

};

**3-Phases Model**

Options()

{

Resolution=1;

};

Plot()

{

Outlier_Model("General",T(5),U(0,4),"t");

Sequence()

{

Boundary("Start 1");

Phase("Construction Phases 1-3b")

{

Phase("Construction Phase 1")

{

V_Sequence("21")

{

R_Date("RTD12280",2089,15)

{

Outlier("General",0.05);

};

Gap(25,4);

R_Date("RTD12281",2077,15)

{

Outlier("General",0.05);

};

Gap(16,2);

R_Date("RTD12282",2038,39)

{

Outlier("General",0.05);

color="brown";

};

Gap(2,1);

R_Date("RTD12039",1981,24)

{

color="brown";

Outlier("General",1);

};

};

V_Sequence("22")

{

R_Date("RTD12283",2129,15)

{

Outlier("General",0.05);

};

Gap(31,4);

R_Date("RTD12040",2050,23)

{

color="orange";

Outlier("General",0.05);

};

};

};

Phase(""Construction Phase 2a"")

{

R_Combine("RTD10490 (9)")

{

color="Green";

R_Date("RTD10490.1 (9)", 1959, 29)

{

Outlier("General",0.05);

};

R_Date("RTD10490.2 (9)", 2040, 38)

{

Outlier("General",0.05);

};

};

R_Date("RTD10489 (8b)", 2005, 18)

{

color="Green";

Outlier("General",0.05);

};

};

Phase("Construction Phase 2b")

{

V_Sequence("10A")

{

R_Date("RTD12284",2062,21)

{

Outlier("General",0.05);

};

Gap(12,2);

R_Date("RTD12285",2118,16)

{

Outlier("General",0.05);

};

Gap(22,4);

R_Date("RTD10643 (10A) outer rings", 2047, 17)

{

color="purple";

Outlier("General",0.05);

};

};

};

Phase("Construction Phase 3b")

{

V_Sequence("13A")

{

R_Date("RTD10666(13A ring#2)",2104,17)

{

Outlier("General",0.05);

};

Gap(16,2);

R_Date("RTD10667(13A ring#18)",2061,17)

{

Outlier("General",0.05);

};

Gap(11,2);

R_Date("RTD10491(13A outer 2 rings)",2031,19)

{

Outlier("General",0.05);

color="Blue";

};

};

};

};

Boundary("3b-3c First boundary");

Phase("Construction Phase 3c")

{

R_Date("RTD10492 (15a)", 2020, 18)

{

color="pink";

Outlier("General",0.05);

};

};

Boundary("3c-5 First boundary");

Boundary("3c-5 Second boundary");

Phase("Construction Phase 5")

{

R_Date("RTD10304 (4b)", 1939, 18)

{

color="Red";

Outlier("General",0.05);

};

V_Sequence("5A")

{

R_Date("RTD10335 (5A) ring#2",1938,28)

{

Outlier("General",0.05);

};

Gap(28,3);

R_Combine("RTD10305 5A outer rings")

{

R_Date("RTD10305",1912,31)

{

Outlier("General",0.05);

};

R_Date("RTD10305.1", 1951, 17)

{

Outlier("General",0.05);

};

color="Red";

};

};

V_Sequence("6B")

{

R_Date("RTD10336 (6B) ring#1",1905,28)

{

Outlier("General",0.05);

};

Gap(12,2);

R_Date("RTD10306 (6B) rings#13-14",1934,28)

{

Outlier("General",0.05);

color="Red";

};

};

V_Sequence("18")

{

R_Date("RTD11464 (18) ring#3",1911,24)

{

Outlier("General",0.05);

};

Gap(12,2);

R_Date("RTD11465 (18) ring#15",1923,24)

{

Outlier("General",0.05);

color="red";

};

};

};

Boundary("End 5");

};

};

**6-Phases Model**

Options()

{

Resolution=1;

};

Plot()

{

Outlier_Model("General",T(5),U(0,4),"t");

Sequence()

{

Boundary("Start 1");

Phase("Construction Phase 1")

{

V_Sequence("21")

{

R_Date("RTD12280",2089,15)

{

Outlier(0.05);

};

Gap(25,4);

R_Date("RTD12281",2077,15)

{

Outlier(0.05);

};

Gap(16,2);

R_Date("RTD12282",2038,39)

{

Outlier(0.05);

color="brown";

};

Gap(2,1);

R_Date("RTD12039",1981,24)

{

color="brown";

Outlier(1);

};

};

V_Sequence("22")

{

R_Date("RTD12283",2129,15)

{

Outlier(0.05);

};

Gap(31,4);

R_Date("RTD12040",2050,23)

{

color="orange";

Outlier(0.05);

};

};

};

Boundary("1-2a");

Phase(""Construction Phase 2a"")

{

R_Combine("RTD10490 (9)")

{

color="Green";

R_Date("RTD10490.1 (9)", 1959, 29)

{

Outlier(0.05);

};

R_Date("RTD10490.2 (9)", 2040, 38)

{

Outlier(0.05);

};

};

R_Date("RTD10489 (8b)", 2005, 18)

{

color="Green";

Outlier(0.05);

};

};

Boundary("2a-2b");

Phase("Construction Phase 2b")

{

V_Sequence("10A")

{

R_Date("RTD12284",2062,21)

{

Outlier(0.05);

};

Gap(12,2);

R_Date("RTD12285",2118,16)

{

Outlier(0.05);

};

Gap(22,4);

R_Date("RTD10643 (10A) outer rings", 2047, 17)

{

color="purple";

Outlier(0.05);

};

};

};

Boundary("2b-3b");

Phase("Construction Phase 3b")

{

V_Sequence("13A")

{

R_Date("RTD10666(13A ring#2)",2104,17)

{

Outlier(0.05);

};

Gap(16,2);

R_Date("RTD10667(13A ring#18)",2061,17)

{

Outlier(0.05);

};

Gap(11,2);

R_Date("RTD10491(13A outer 2 rings)",2031,19)

{

Outlier(0.05);

color="Blue";

};

};

};

Boundary("3b-3c");

Phase("Construction Phase 3c")

{

R_Date("RTD10492 (15a)", 2020, 18)

{

color="Pink";

Outlier(0.05);

};

};

Boundary("3c-5 First boundary");

Boundary("3c-5 Second boundary");

Phase("Construction Phase 5")

{

R_Date("RTD10304 (4b)", 1939, 18)

{

color="Red";

Outlier(0.05);

};

V_Sequence("5A")

{

R_Date("RTD10335 (5A) ring#2",1938,28)

{

Outlier(0.05);

};

Gap(28,3);

R_Combine("RTD10305 5A outer rings")

{

R_Date("RTD10305",1912,31)

{

Outlier(0.05);

};

R_Date("RTD10305.1", 1951, 17)

{

Outlier(0.05);

};

color="Red";

};

};

V_Sequence("6B")

{

R_Date("RTD10336 (6B) ring#1",1905,28)

{

Outlier(0.05);

};

Gap(12,2);

R_Date("RTD10306 (6B) rings#13-14",1934,28)

{

Outlier(0.05);

color="Red";

};

};

V_Sequence("18")

{

R_Date("RTD11464 (18) ring#3",1911,24)

{

Outlier(0.05);

};

Gap(12,2);

R_Date("RTD11465 (18) ring#15",1923,24)

{

Outlier(0.05);

color="red";

};

};

};

Boundary("End 5");

};

};

**2-Phases Model, including offset**

Options()

{

Resolution=1;

};

Plot()

{

Outlier_Model("General",T(5),U(0,4),"t");

Delta_R(11,5);

Sequence()

{

Boundary("Start 1-3c");

Phase("Construction Phases 1-3c")

{

V_Sequence("21")

{

R_Date("RTD12280",2089,15)

{

Outlier(0.05);

};

Gap(25,4);

R_Date("RTD12281",2077,15)

{

Outlier(0.05);

};

Gap(16,2);

R_Date("RTD12282",2038,39)

{

color="brown";

Outlier(0.05);

};

Gap(2,1);

R_Date("RTD12039",1981,24)

{

color="brown";

Outlier(1);

};

};

V_Sequence("22")

{

R_Date("RTD12283",2129,15)

{

Outlier(0.05);

};

Gap(31,4);

R_Date("RTD12040",2050,23)

{

color="orange";

Outlier(0.05);

};

};

R_Combine("RTD10490 (9)")

{

color="Green";

R_Date("RTD10490.1 (9)", 1959, 29)

{

Outlier(0.05);

};

R_Date("RTD10490.2 (9)", 2040, 38)

{

Outlier(0.05);

};

};

R_Date("RTD10489 (8b)", 2005, 18)

{

color="Green";

Outlier(0.05);

};

Delta_R(0,0);

V_Sequence("13A")

{

R_Date("RTD10666(13A ring#2)",2104,17)

{

Outlier(0.05);

};

Gap(16,2);

R_Date("RTD10667(13A ring#18)",2061,17)

{

Outlier(0.05);

};

Gap(11,2);

R_Date("RTD10491(13A outer 2 rings)",2031,19)

{

Outlier(0.05);

color="Blue";

};

};

Delta_R(11,5);

V_Sequence("10A")

{

R_Date("RTD12284",2062,21)

{

Outlier(0.05);

};

Gap(12,2);

R_Date("RTD12285",2118,16)

{

Outlier(0.05);

};

Gap(22,4);

R_Date("RTD10643 (10A) outer rings", 2047, 17)

{

color="purple";

Outlier(0.05);

};

};

R_Date("RTD10492 (15a)", 2020, 18)

{

color="pink";

Outlier(0.05);

};

};

Boundary("3c-5 First boundary");

Boundary("3c-5 Second boundary");

Phase("Construction Phase 5")

{

R_Date("RTD10304 (4b)", 1939, 18)

{

color="Red";

Outlier(0.05);

};

V_Sequence("5A")

{

R_Date("RTD10335 (5A) ring#2",1938,28)

{

Outlier(0.05);

};

Gap(28,3);

R_Combine("RTD10305 5A outer rings")

{

R_Date("RTD10305",1912,31)

{

Outlier(0.05);

};

R_Date("RTD10305.1", 1951, 17)

{

Outlier(0.05);

};

color="Red";

};

};

Delta_R(0,0);

V_Sequence("6B")

{

R_Date("RTD10336 (6B) ring#1",1905,28)

{

Outlier(0.05);

};

Gap(12,2);

R_Date("RTD10306 (6B) rings#13-14",1934,28)

{

Outlier(0.05);

color="Red";

};

};

Delta_R(11,5);

V_Sequence("18")

{

R_Date("RTD11464 (18) ring#3",1911,24)

{

Outlier(0.05);

};

Gap(12,2);

R_Date("RTD11465 (18) ring#15",1923,24)

{

Outlier(0.05);

color="red";

};

};

};

Boundary("End 5");

};

};

**3-Phases Model, including offset**

Options()

{

Resolution=1;

};

Plot()

{

Outlier_Model("General",T(5),U(0,4),"t");

Delta_R(11,5);

Sequence()

{

Boundary("Start 1");

Phase("Construction Phases 1-3b")

{

Phase("Construction Phase 1")

{

V_Sequence("21")

{

R_Date("RTD12280",2089,15)

{

Outlier("General",0.05);

};

Gap(25,4);

R_Date("RTD12281",2077,15)

{

Outlier("General",0.05);

};

Gap(16,2);

R_Date("RTD12282",2038,39)

{

Outlier("General",0.05);

color="brown";

};

Gap(2,1);

R_Date("RTD12039",1981,24)

{

color="brown";

Outlier("General",1);

};

};

V_Sequence("22")

{

R_Date("RTD12283",2129,15)

{

Outlier("General",0.05);

};

Gap(31,4);

R_Date("RTD12040",2050,23)

{

color="orange";

Outlier("General",0.05);

};

};

};

Phase(""Construction Phase 2a"")

{

R_Combine("RTD10490 (9)")

{

color="Green";

R_Date("RTD10490.1 (9)", 1959, 29)

{

Outlier("General",0.05);

};

R_Date("RTD10490.2 (9)", 2040, 38)

{

Outlier("General",0.05);

};

};

R_Date("RTD10489 (8b)", 2005, 18)

{

color="Green";

Outlier("General",0.05);

};

};

Phase("Construction Phase 2b")

{

V_Sequence("10A")

{

R_Date("RTD12284",2062,21)

{

Outlier("General",0.05);

};

Gap(12,2);

R_Date("RTD12285",2118,16)

{

Outlier("General",0.05);

};

Gap(22,4);

R_Date("RTD10643 (10A) outer rings", 2047, 17)

{

color="purple";

Outlier("General",0.05);

};

};

};

Phase("Construction Phase 3b")

{

Delta_R(0,0);

V_Sequence("13A")

{

R_Date("RTD10666(13A ring#2)",2104,17)

{

Outlier("General",0.05);

};

Gap(16,2);

R_Date("RTD10667(13A ring#18)",2061,17)

{

Outlier("General",0.05);

};

Gap(11,2);

R_Date("RTD10491(13A outer 2 rings)",2031,19)

{

Outlier("General",0.05);

color="Blue";

};

};

};

};

Boundary("3b-3c First boundary");

Phase("Construction Phase 3c")

{

Delta_R(11,5);

R_Date("RTD10492 (15a)", 2020, 18)

{

color="pink";

Outlier("General",0.05);

};

};

Boundary("3c-5 First boundary");

Boundary("3c-5 Second boundary");

Phase("Construction Phase 5")

{

R_Date("RTD10304 (4b)", 1939, 18)

{

color="Red";

Outlier("General",0.05);

};

V_Sequence("5A")

{

R_Date("RTD10335 (5A) ring#2",1938,28)

{

Outlier("General",0.05);

};

Gap(28,3);

R_Combine("RTD10305 5A outer rings")

{

R_Date("RTD10305",1912,31)

{

Outlier("General",0.05);

};

R_Date("RTD10305.1", 1951, 17)

{

Outlier("General",0.05);

};

color="Red";

};

};

Delta_R(0,0);

V_Sequence("6B")

{

R_Date("RTD10336 (6B) ring#1",1905,28)

{

Outlier("General",0.05);

};

Gap(12,2);

R_Date("RTD10306 (6B) rings#13-14",1934,28)

{

Outlier("General",0.05);

color="Red";

};

};

Delta_R(11,5);

V_Sequence("18")

{

R_Date("RTD11464 (18) ring#3",1911,24)

{

Outlier("General",0.05);

};

Gap(12,2);

R_Date("RTD11465 (18) ring#15",1923,24)

{

Outlier("General",0.05);

color="red";

};

};

};

Boundary("End 5");

};

};

**4-Phases Model, including offset**

Options()

{

Resolution=1;

};

Plot()

{

Outlier_Model("General",T(5),U(0,4),"t");

Delta_R(11,5);

Sequence()

{

Boundary("Start 1");

Phase("Construction Phase 1")

{

V_Sequence("21")

{

R_Date("RTD12280",2089,15)

{

Outlier("General",0.05);

};

Gap(25,4);

R_Date("RTD12281",2077,15)

{

Outlier("General",0.05);

};

Gap(16,2);

R_Date("RTD12282",2038,39)

{

Outlier("General",0.05);

color="brown";

};

Gap(2,1);

R_Date("RTD12039",1981,24)

{

color="brown";

Outlier("General",1);

};

};

V_Sequence("22")

{

R_Date("RTD12283",2129,15)

{

Outlier("General",0.05);

};

Gap(31,4);

R_Date("RTD12040",2050,23)

{

color="orange";

Outlier("General",0.05);

};

};

};

Boundary("1-2");

Phase("Construction Phases 2-3b")

{

Phase(""Construction Phase 2a"")

{

R_Combine("RTD10490 (9)")

{

color="Green";

R_Date("RTD10490.1 (9)", 1959, 29)

{

Outlier("General",0.05);

};

R_Date("RTD10490.2 (9)", 2040, 38)

{

Outlier("General",0.05);

};

};

R_Date("RTD10489 (8b)", 2005, 18)

{

color="Green";

Outlier("General",0.05);

};

};

Phase("Construction Phase 2b")

{

V_Sequence("10A")

{

R_Date("RTD12284",2062,21)

{

Outlier("General",0.05);

};

Gap(12,2);

R_Date("RTD12285",2118,16)

{

Outlier("General",0.05);

};

Gap(22,4);

R_Date("RTD10643 (10A) outer rings", 2047, 17)

{

color="purple";

Outlier("General",0.05);

};

};

};

Phase("Construction Phase 3b")

{

Delta_R(0,0);

V_Sequence("13A")

{

R_Date("RTD10666(13A ring#2)",2104,17)

{

Outlier("General",0.05);

};

Gap(16,2);

R_Date("RTD10667(13A ring#18)",2061,17)

{

Outlier("General",0.05);

};

Gap(11,2);

R_Date("RTD10491(13A outer 2 rings)",2031,19)

{

Outlier("General",0.05);

color="Blue";

};

};

};

};

Boundary("3b-3c First boundary");

Delta_R(11,5);

Phase("Construction Phase 3c")

{

R_Date("RTD10492 (15a)", 2020, 18)

{

color="pink";

Outlier("General",0.05);

};

};

Boundary("3c-5 First boundary");

Boundary("3c-5 Second boundary");

Phase("Construction Phase 5")

{

R_Date("RTD10304 (4b)", 1939, 18)

{

color="Red";

Outlier("General",0.05);

};

V_Sequence("5A")

{

R_Date("RTD10335 (5A) ring#2",1938,28)

{

Outlier("General",0.05);

};

Gap(28,3);

R_Combine("RTD10305 5A outer rings")

{

R_Date("RTD10305",1912,31)

{

Outlier("General",0.05);

};

R_Date("RTD10305.1", 1951, 17)

{

Outlier("General",0.05);

};

color="Red";

};

};

Delta_R(0,0);

V_Sequence("6B")

{

R_Date("RTD10336 (6B) ring#1",1905,28)

{

Outlier("General",0.05);

};

Gap(12,2);

R_Date("RTD10306 (6B) rings#13-14",1934,28)

{

Outlier("General",0.05);

color="Red";

};

};

Delta_R(11,5);

V_Sequence("18")

{

R_Date("RTD11464 (18) ring#3",1911,24)

{

Outlier("General",0.05);

};

Gap(12,2);

R_Date("RTD11465 (18) ring#15",1923,24)

{

Outlier("General",0.05);

color="red";

};

};

};

Boundary("End 5");

};

};

**6-Phases Model, including offset**

Options()

{

Resolution=1;

};

Plot()

{

Outlier_Model("General",T(5),U(0,4),"t");

Delta_R(11,5);

Sequence()

{

Boundary("Start 1");

Phase("Construction Phase 1")

{

V_Sequence("21")

{

R_Date("RTD12280",2089,15)

{

Outlier(0.05);

};

Gap(25,4);

R_Date("RTD12281",2077,15)

{

Outlier(0.05);

};

Gap(16,2);

R_Date("RTD12282",2038,39)

{

Outlier(0.05);

color="brown";

};

Gap(2,1);

R_Date("RTD12039",1981,24)

{

color="brown";

Outlier(1);

};

};

V_Sequence("22")

{

R_Date("RTD12283",2129,15)

{

Outlier(0.05);

};

Gap(31,4);

R_Date("RTD12040",2050,23)

{

color="orange";

Outlier(0.05);

};

};

};

Boundary("1-2a");

Phase(""Construction Phase 2a"")

{

R_Combine("RTD10490 (9)")

{

color="Green";

R_Date("RTD10490.1 (9)", 1959, 29)

{

Outlier(0.05);

};

R_Date("RTD10490.2 (9)", 2040, 38)

{

Outlier(0.05);

};

};

R_Date("RTD10489 (8b)", 2005, 18)

{

color="Green";

Outlier(0.05);

};

};

Boundary("2a-2b");

Phase("Construction Phase 2b")

{

V_Sequence("10A")

{

R_Date("RTD12284",2062,21)

{

Outlier(0.05);

};

Gap(12,2);

R_Date("RTD12285",2118,16)

{

Outlier(0.05);

};

Gap(22,4);

R_Date("RTD10643 (10A) outer rings", 2047, 17)

{

color="purple";

Outlier(0.05);

};

};

};

Boundary("2b-3b");

Phase("Construction Phase 3b")

{

Delta_R(0,0);

V_Sequence("13A")

{

R_Date("RTD10666(13A ring#2)",2104,17)

{

Outlier(0.05);

};

Gap(16,2);

R_Date("RTD10667(13A ring#18)",2061,17)

{

Outlier(0.05);

};

Gap(11,2);

R_Date("RTD10491(13A outer 2 rings)",2031,19)

{

Outlier(0.05);

color="Blue";

};

};

};

Boundary("3b-3c");

Phase("Construction Phase 3c")

{

Delta_R(11,5);

R_Date("RTD10492 (15a)", 2020, 18)

{

color="Pink";

Outlier(0.05);

};

};

Boundary("3c-5 First boundary");

Boundary("3c-5 Second boundary");

Phase("Construction Phase 5")

{

R_Date("RTD10304 (4b)", 1939, 18)

{

color="Red";

Outlier(0.05);

};

V_Sequence("5A")

{

R_Date("RTD10335 (5A) ring#2",1938,28)

{

Outlier(0.05);

};

Gap(28,3);

R_Combine("RTD10305 5A outer rings")

{

R_Date("RTD10305",1912,31)

{

Outlier(0.05);

};

R_Date("RTD10305.1", 1951, 17)

{

Outlier(0.05);

};

color="Red";

};

};

Delta_R(0,0);

V_Sequence("6B")

{

R_Date("RTD10336 (6B) ring#1",1905,28)

{

Outlier(0.05);

};

Gap(12,2);

R_Date("RTD10306 (6B) rings#13-14",1934,28)

{

Outlier(0.05);

color="Red";

};

};

Delta_R(11,5);

V_Sequence("18")

{

R_Date("RTD11464 (18) ring#3",1911,24)

{

Outlier(0.05);

};

Gap(12,2);

R_Date("RTD11465 (18) ring#15",1923,24)

{

Outlier(0.05);

color="red";

};

};

};

Boundary("End 5");

};

};
